# Conditional Creation and Rescue of *Nipbl*-Deficiency in Mice Reveals Multiple Determinants of Risk for Congenital Heart Defects

**DOI:** 10.1371/journal.pbio.2000197

**Published:** 2016-09-08

**Authors:** Rosaysela Santos, Shimako Kawauchi, Russell E. Jacobs, Martha E. Lopez-Burks, Hojae Choi, Jamie Wikenheiser, Benedikt Hallgrimsson, Heather A. Jamniczky, Scott E. Fraser, Arthur D. Lander, Anne L. Calof

**Affiliations:** 1 Department of Developmental and Cell Biology, University of California, Irvine, California, United States of America; 2 Center for Complex Biological Systems, University of California, Irvine, California, United States of America; 3 Biological Imaging Center, Beckman Institute, California Institute of Technology, Pasadena, California, United States of America; 4 Department of Anatomy and Neurobiology, University of California, Irvine, California, United States of America; 5 Department of Cell Biology and Anatomy, University of Calgary, Calgary, Alberta, Canada; 6 Departments of Biology and Bioengineering, University of Southern California, Los Angeles, California, United States of America; University of Pittsburgh, United States

## Abstract

Elucidating the causes of congenital heart defects is made difficult by the complex morphogenesis of the mammalian heart, which takes place early in development, involves contributions from multiple germ layers, and is controlled by many genes. Here, we use a conditional/invertible genetic strategy to identify the cell lineage(s) responsible for the development of heart defects in a *Nipbl*-deficient mouse model of Cornelia de Lange Syndrome, in which global yet subtle transcriptional dysregulation leads to development of atrial septal defects (ASDs) at high frequency. Using an approach that allows for recombinase-mediated creation or rescue of *Nipbl* deficiency in different lineages, we uncover complex interactions between the cardiac mesoderm, endoderm, and the rest of the embryo, whereby the risk conferred by genetic abnormality in any one lineage is modified, in a surprisingly non-additive way, by the status of others. We argue that these results are best understood in the context of a model in which the risk of heart defects is associated with the adequacy of early progenitor cell populations relative to the sizes of the structures they must eventually form.

## Author Summary

Congenital heart defects, the most common birth defect, are thought mainly to arise through interactions among multiple genes. We studied atrial septal defects (a common type of heart defect) in a mouse model of Cornelia de Lange Syndrome, in which loss of one copy of the *Nipbl* gene produces frequent developmental abnormalities. By causing subtle dysregulation of the expression of many hundreds of genes, *Nipbl* haploinsufficiency serves as a model for the polygenic origins of common birth defects. We used an improved genetic technology to separately create or rescue deficiency for *Nipbl* within the major cardiogenic tissue lineages (lineages that contribute to or control the morphogenesis of the developing heart). Unexpectedly, we found that risk for developing atrial septal defects could not be mapped to any single one of these cardiogenic lineages, but rather was determined by non-additive interactions between these lineages and the rest of the body. Intriguingly, being *Nipbl*-deficient in the rest of the body reduced the risk conferred by being *Nipbl*-deficient in either of two distinct cardiogenic lineages. We hypothesize that this effect is driven by developmental coupling between body size and heart size, with defects arising when progenitor cells cannot be provided fast enough to meet the requirements imposed on the heart by other growing tissues. To our knowledge, this is the first genetic demonstration that major risk factors for heart defects are likely to lie outside of the heart itself.

## Introduction

Congenital heart defects (CHDs) are the most common of human birth defects, and a leading cause of perinatal morbidity and mortality [1]. The genetics of CHDs are complex, with only a fraction being associated with chromosomal abnormalities or Mendelian developmental syndromes [2–4]. As with most human birth defects, the majority of CHDs are non-syndromic (isolated), and appear to be multifactorial and most likely polygenic [5–8].

Cornelia de Lange Syndrome (CdLS) is a multisystem birth defects disorder characterized by craniofacial abnormalities, developmental delay in growth and maturation, neurological deficits and intellectual disability, limb abnormalities (particularly of the arms and hands), as well as defects in the visual, auditory, gastrointestinal, genitourinary, and cardiopulmonary systems [9–13]. CHDs are seen in about 30% of individuals with CdLS [14,15], with structural defects of the ventricular septum (VSD) and atrial septum (ASD) being among the most common.

The genetic cause of CdLS is, in most cases, haploinsufficiency for *NIPBL* (Nipped-B-homologue), which encodes a ubiquitous protein that regulates the loading of cohesin onto chromosomes [16,17]. Cohesin, a multiprotein complex originally identified by its role in sister chromatid cohesion, is now understood to play critical roles in the regulation of gene expression [18–20]. In support of this view, both CdLS patient cell lines and animal models of *Nipbl* haploinsufficiency display small but significant changes in the expression of up to 1,000 or more genes, in essentially every tissue [21–24]. Studies in mouse and zebrafish models suggest that these gene expression changes—most too small to have phenotypic consequences individually—collectively cause the structural and functional defects observed in CdLS [23,25–27]. CdLS and related “cohesinopathies” [26] thus exemplify an emerging class of genetic disorders—recently termed “transcriptomopathies” [27]—in which additive or synergistic effects of quantitative variation in the levels of multiple gene products lie at the root of developmental abnormalities. Such disorders provide a unique window into the kinds of multifactorial interactions that likely underlie polygenic traits, including the majority of complex developmental disease.

Among features of CdLS that are phenocopied in *Nipbl-*deficient animal models are CHDs [22,23]. In particular, mouse models display a high frequency of ASD, which are both common in CdLS and among the most common CHDs in non-syndromic settings [28,29]. Elucidating the processes that lead to CHDs in such mouse models could potentially provide valuable insight into the multifactorial causation of birth defects. Yet the fact that gene expression is globally affected in such animals also makes it difficult to know a priori in which cell types, or at what developmental stages, to look for such processes. For heart defects, this is an especially challenging issue, as development of the heart involves communication and interaction among many cell types, in multiple tissues, across many stages of development [30–32].

Here, we address this problem by exploiting a new allelic series (*Nipbl*^*FLEX*^), in which cells, and mice, can be toggled between wildtype and mutant conformations of *Nipbl* using Flip-Excision (FLEX) technology [33]. We use this strategy both to create and rescue *Nipbl*-deficiency in multiple lineages during early heart development, including the cardiogenic mesoderm, endoderm, and neural crest. Interestingly, the data do not support the assignment of CHD risk to a single cardiac lineage, but rather suggest that the cardiogenic mesoderm, the endoderm, and other lineages all participate, interacting in surprisingly non-additive, and even antagonistic, ways. We relate these findings to morphological and gene expression changes that occur in early *Nipbl*-deficient hearts, and propose a model in which defects arise when the ability to generate cardiac tissue during very early stages fails to keep up with the demands imposed by final heart size.

## Results

### *Nipbl*-Deficient Mice Display Heart Abnormalities throughout Embryonic Development

**Fig 1:**
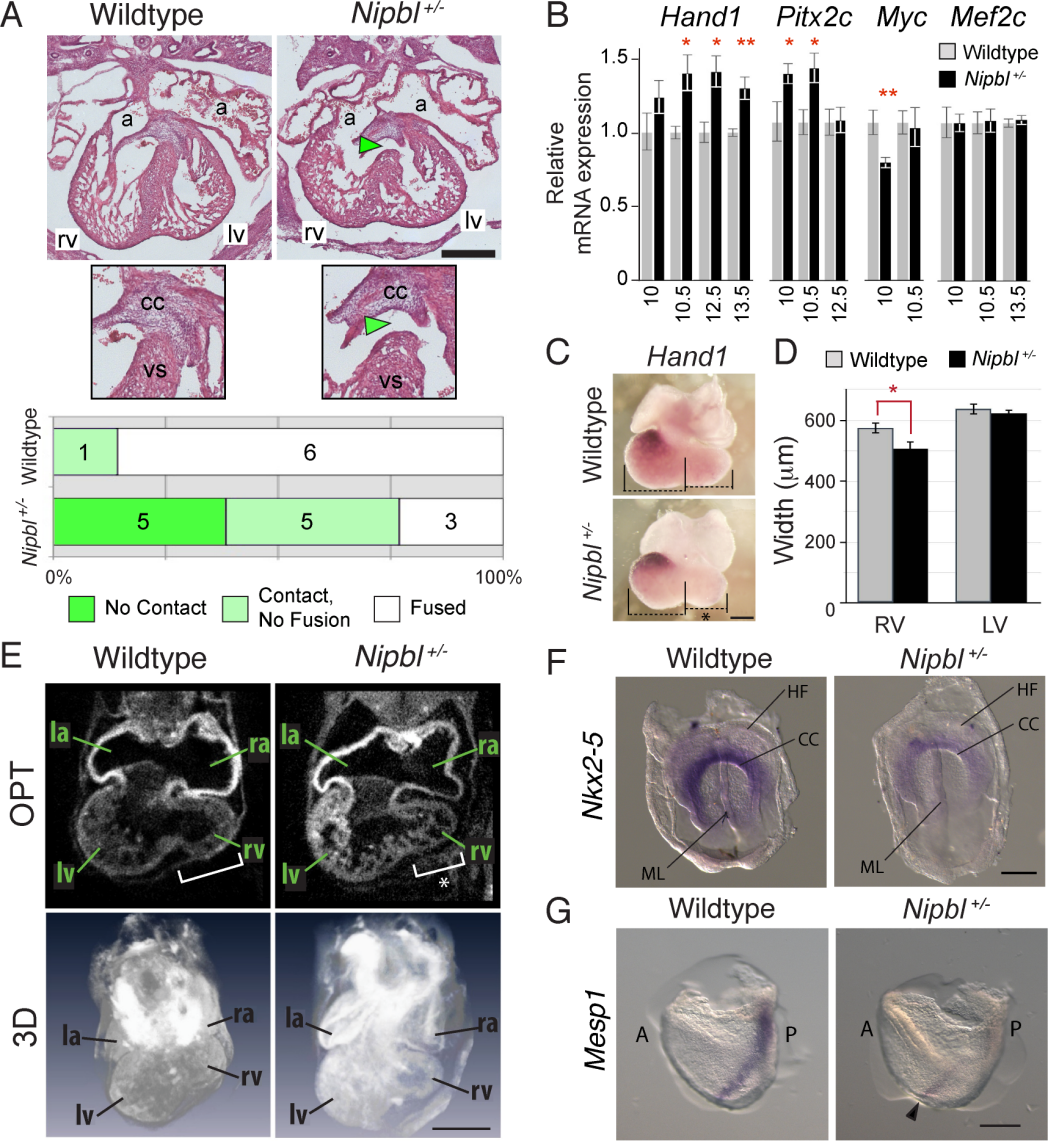
*Nipbl*-deficient mice show abnormalities throughout heart development. A. Hematoxylin and Eosin Y (H&E)-stained sectioned hearts illustrating delay in fusion of ventricular septum (VS) with cardiac cushion (CC) in *Nipbl*^*+/-*^ mice at E13.5. The majority of wildtype hearts display a fully-developed VS fused with the CC (left panels); 1/7 (14%) display a mild phenotype (contact between the CC and VS, but no fusion). 77% (10/13) of *Nipbl*^*+/-*^ hearts show a defect in VS fusion: 38% (5/13) have a complete lack of contact between VS and CC (right panels, green arrowhead); 38% (5/13) have a milder phenotype (contact between the CC and VS, but no fusion). Frequencies of each defect are plotted in histogram. *p* = 0.0166, Fisher’s Exact test (*Nipbl*^*+/-*^, 10/13 versus wildtype, 1/7). a, atrium; lv, left ventricle; rv, right ventricle; vs, ventricular septum. Size bar = 400 μm. B. Quantitative reverse transcription PCR (qRT-PCR) was performed on hearts at different stages between E10 and E13.5. Relative expression of *Hand1*, *Pitx2*, and *cMyc*, but not *Mef2c*, differed significantly in *Nipbl*^*+/-*^ versus wildtype hearts at indicated stages. Histogram shows mean ± standard error of the mean (SEM) of relative gene expression. *p < 0.05, ** *p* < 0.01, Student’s *t* test (S1 Data). C. In situ hybridization (ISH) for *Hand1* at E10.5 showing similar pattern of expression between *Nipbl*^*+/-*^ and wildtype hearts. A discrepancy in the size of the developing right ventricle of *Nipbl*^*+/-*^ hearts is observable (black asterisk). Size bar = 400 μm. D. Left and right ventricle diameters were measured from whole mount E10.5 heart images using Axiovision software (Zeiss). Histograms plot means (± SEM) for right and left ventricle diameters of *Nipbl*^*+/-*^ hearts (*n* = 5) and stage-matched wildtype littermate controls (*n* = 7). * *p* < 0.05, Student’s *t* test. E. OPT images showing sections and 3-D reconstructions of E10.5 hearts, illustrating that right ventricles are smaller in E10.5 *Nipbl*^*+/-*^ hearts (white asterisk). Size bar = 400 μm. F. ISH at cardiac crescent stage (E7.5) shows a reduction in *Nkx2-5* expression in the cardiac crescent (CC) of *Nipbl*^*+/-*^ mice (*n* = 2) compared to wildtype littermates (*n* = 3). ML, midline; HF, headfold. Scale bar: 200 μm. G. ISH at primitive streak stage (E6.5) shows a drastic reduction in *Mesp1* expression in *Nipbl*^*+/-*^ embryos (*n* = 5, black arrowhead) compared to wildtype littermates (*n* = 5). A, anterior; P, posterior. Scale bar: 200 μm.

In a previous study of a CdLS mouse model of *Nipbl* haploinsufficiency (*Nipbl*^+/-^ mice), we reported that about half of *Nipbl*^+/-^ mice display defects in closure of the atrial septum (ASD) between gestational days 15.5 and 17.5 (E15.5–E17.5) [22]. Because many of the nearly 100 genes that have been linked to the production of cardiac septal defects [34,35] influence heart development through actions at early embryonic stages, long before cardiac septa form, we also examined heart development in *Nipbl*^+/-^ mice at successively earlier developmental stages to determine whether earlier structural abnormalities could be found. Interestingly, even though E17.5 *Nipbl*^+/-^ mice do not usually display defects of the ventricular septum, we found evidence for earlier abnormalities in ventricular septation. In particular, at E13.5, 77% of *Nipbl*^*+/-*^ hearts showed incomplete fusion or complete lack of contact of the developing ventricular septum with the cardiac cushion, compared to 14% of wildtype hearts (Fig 1A).

Studies of *Nipbl*-deficient tissues and cells from *Drosophila*, mouse, zebrafish, and man all indicate that the means by which *Nipbl*-deficiency causes birth defects is by subtly mis-regulating the expression of large numbers of genes [21–25]. Accordingly, we screened *Nipbl*^*+/-*^ hearts for abnormalities in the expression of genes known to be involved in cardiac septation. Using quantitative reverse transcription PCR (qRT-PCR) as a rapid screen, we assessed expression of 29 such genes (S1 Table) from E10–10.5, when all four heart chambers have formed and septation has just begun; through E13.5, when ventricular septation is mostly complete [30].

Three genes (*Hand1*, *Pitx2c*, and *cMyc*) showed consistent changes in *Nipbl*^*+/-*^ hearts (Fig 1B and S1 Fig). Levels of *Hand1* and *Pitx2* were increased (by up to 40%), while *cMyc* was reduced by ~25% at E10. No statistically significant changes were detected for other tested genes (except *Nipbl* itself which, as expected, was reduced in *Nipbl*^*+/-*^ hearts [by ~50%; S1 Fig]). One example of an unaffected gene (*Mef2C*, which plays a critical early role in vertebrate cardiovascular development [36]) is shown in Fig 1B.

These relatively small alterations of gene expression are typical of transcriptional effects in *Nipbl*-deficient animal models and CdLS cell lines [21,22,24,25]. In the case of *cMyc*, the change is likely a direct effect, as *cMyc* down-regulation is a hallmark of *Nipbl*-deficiency in almost every cell type and organism examined to date [21–24,37].

*Hand1* and *Pitx2* are genes that both play a role in left-right asymmetry. *Pitx2* encodes a homeobox transcription factor critical for left-right patterning of the entire body, and is preferentially expressed in left-sided cardiac structures from as early as cardiac crescent stage [38], and *Pitx2* mutations have been associated with cardiac defects, including septal defects [39–42]. *Hand1* encodes a basic helix-loop-helix transcription factor that is preferentially expressed in the left ventricle, and is important for its development [43–45].

Because both *Pitx2* and *Hand1* are associated with left heart structures, we wondered whether their elevated levels in E10–10.5 *Nipbl*^*+/-*^ hearts was due to a change in gene regulation, or a change in the proportions of left-sided versus right-sided tissue. Close inspection of hearts in which *Hand1* was visualized by in situ hybridization (ISH; Fig 1C and 1D), as well as hearts imaged by optical projection tomography (OPT; Fig 1E), supports the latter explanation. As shown in Fig 1C, E10.5 *Nipbl*^*+/-*^ hearts display a relatively normal pattern and intensity of *Hand1* expression (by ISH), but the right ventricle is abnormally small. This could be demonstrated consistently across embryos (Fig 1D) and was also evident in optical cross-sections through the heart (Fig 1E).

These results suggest that the origins of heart defects in *Nipbl*^*+/-*^ mice lie earlier in development, in events that determine the relative amounts of left and right tissue that contribute to the heart. Among the most important genes upstream of *Hand1* and *Pitx2* is *Nkx2-5* [46,47], which regulates many heart development genes [43]. *Nkx2-5* can be detected as early as E7.5 in the cardiac crescent, the collection of cardiogenic mesodermal cells that later coalesce to form the early heart tube [48]. We used ISH to examine *Nkx2-5* expression at this stage (Fig 1F) and found that while the morphology of the cardiac crescent appears normal in *Nipbl* ^*+/-*^ embryos, the strength of hybridization signal for *Nkx2-5* is noticeably lower than in wildtype littermates, suggesting that either fewer cardiac progenitor cells are present, or these cells express *Nkx2-5* at a reduced level.

The earliest-known marker of cardiogenic mesoderm is *Mesp1*, which acts upstream of *Nkx2*-5 to drive initial specification of cardiac stem/progenitor cells [49–52]. *Mesp1* first appears in the lateral plate mesoderm at the primitive streak stage, about E6.5. By ISH, we observe a dramatic reduction in *Mesp1* in *Nipbl*^*+/-*^ embryos at E6.5 (Fig 1G) which we suspect, given the critical role of *Mesp1* in cardiac development, is probably due to delayed onset of *Mesp1* expression, rather than a loss of the capacity to express *Mesp1*.

Overall, these data indicate that heart development is abnormal in *Nipbl*^*+/-*^ embryos from the earliest developmental times at which cardiogenic tissue is formed. These abnormalities are reflected in reduced expression of key transcription factors at appropriate stages, and delayed growth and development of right-ventricular structures and the interventricular septum.

### Generation of a Conditional/Invertible *Nipbl* Allelic Series Permits Tissue-Specific Creation and Rescue of *Nipbl* Expression Deficiency

Elucidating the mechanisms that underlie structural defects in *Nipbl*-deficient hearts is complicated by the fact that *Nipbl* is a ubiquitously expressed gene, and *Nipbl*-deficiency undoubtedly affects gene expression in every tissue of the embryo. Even given the data in Fig 1, which show changes in the expression of cardiac developmental genes at multiple stages of heart development, it is possible that the actual cause of ASDs lies elsewhere, especially since morphogenesis of the heart involves coordinated interaction among multiple cell types and tissue lineages. For example, the proper specification and patterning of cardiac mesoderm requires interaction with endodermal cells that provide a substrate along which cardiac progenitors migrate to form the cardiac crescent [31]. Indeed, in zebrafish, gene expression abnormalities in the endoderm appear to be the direct cause of some—but not all—cardiac abnormalities that accompany *Nipbl* deficiency [23]. Another lineage that contributes substantially to the heart is the neural crest, which although ectodermal in origin, contributes to the cardiac cushion and outflow tract and has been implicated in the etiology of septal defects [34].

**Fig 2:**
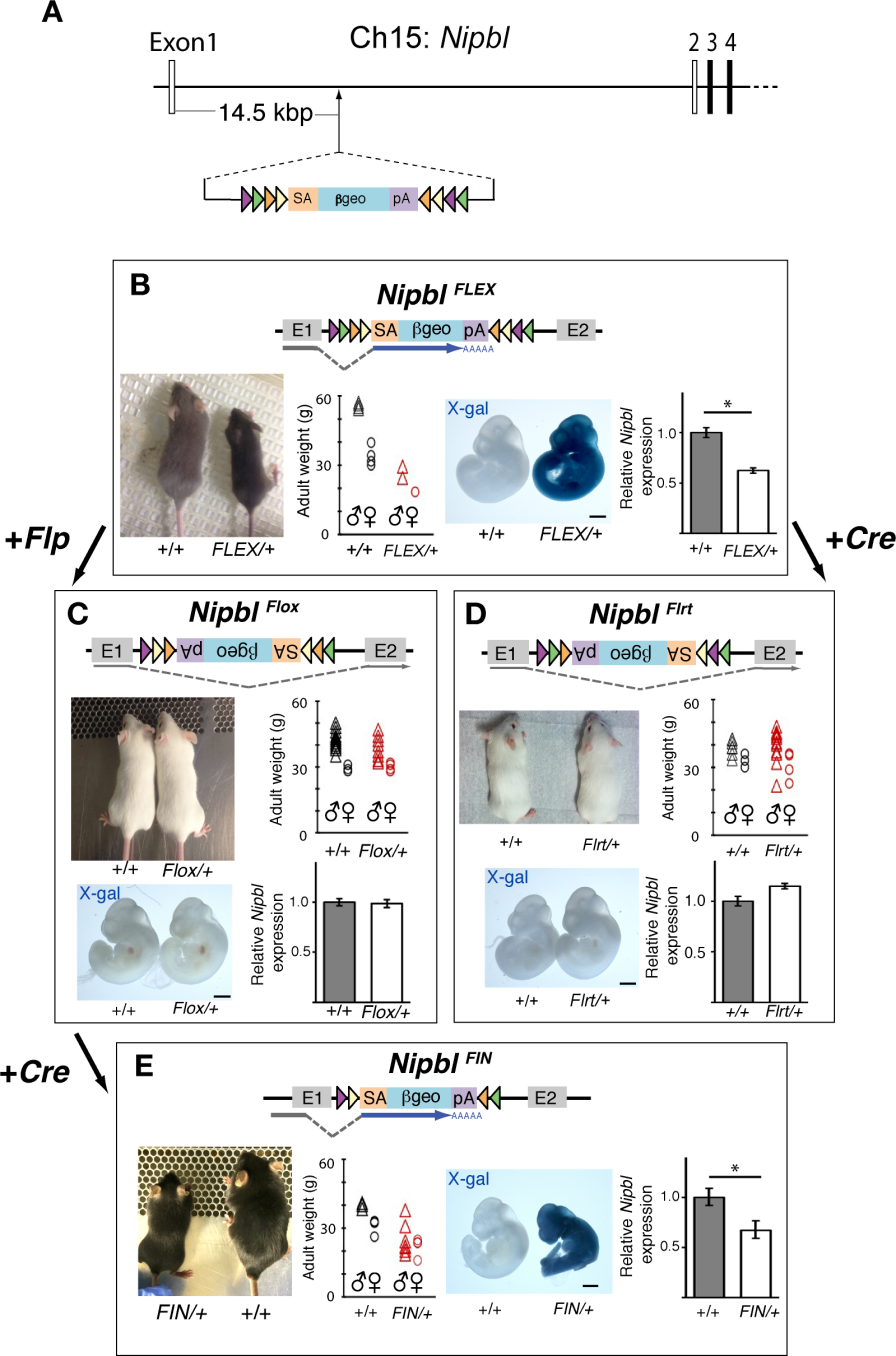
*FLEX* alleles allow successive toggling between mutant and wildtype genotypes and phenotypes. A. Schematic of EUCE313f02 (*Nipbl*^*FLEX*^) allele from which the *Nipbl*^*FLEX/+*^ mouse line and allelic series are derived. The rsFlp-Rosa-βgeo cassette is inserted 14.5 kbp downstream of *Nipbl* Exon 1 on Chromosome 15. B. In the *Nipbl*^*FLEX*^ allele, the splice acceptor (SA) in the cassette traps *Nipbl* expression, resulting in termination of *Nipbl* expression after exon 1 and expression of the *β-geo* reporter for the trapped null allele. Adult *Nipbl*^*FLEX/+*^ mice are smaller than wildtype littermates: Image is of 4-wk-old male littermates. Scatter plot shows weights of 12-wk-old *Nipbl*^*FLEX/+*^ mice (red, *n* = 3: 1 female, 2 males) and wildtype littermates (black, *n* = 8: 4 females, 4 males) from 3 litters. Ubiquitous expression of *β-geo* was detected by X-gal staining in E10.5 *Nipbl*^*FLEX/+*^ embryos. Histogram shows mean ± SEM of relative *Nipbl* expression, assessed by qRT-PCR, in kidneys of E17.5 *Nipbl*^*FLEX/+*^ (*n* = 8) and wildtype littermates (*n* = 6); asterisk: *p* < 0.05 by Student’s *t* test. C. Mating *Nipbl*^*FLEX/+*^ mice with mice carrying universal Flp recombinase inverts the SA-*βgeo*-pA at heterotypic recognition targets (frt and F3 sites) and simultaneously excises cognate recognition sites, resulting in progeny carrying the *Nipbl*^*Flox/+*^ allele. Inversion allows normal splicing between the endogenous *Nipbl* splice sites (Exon 1 to Exon 2), thereby yielding a phenotypically wildtype allele. *Nipbl*^*Flox/+*^ mice are similar to wildtype littermates in size: Image is of 3-wk-old male littermates; scatter plot shows weights of 11-wk-old *Nipbl*^*Flox/+*^ mice (red, *n* = 18: 4 female; 14 male) compared to wildtype littermates (black, *n* = 19: 4 female; 15 male) from 5 litters. Expression of *β-geo* is not detected by X-gal staining in E10.5 *Nipbl*^*Flox/+*^ embryos. Histogram shows qRT-PCR analysis of relative *Nipbl* expression in brain tissue of E17.5 in *Nipbl*^*Flox/+*^ (*n* = 8) versus wildtype littermates (*n* = 7), plotted as in B; *p* > 0.05, Student’s *t* test. D. Mating *Nipbl*^*FLEX/+*^ mice with mice carrying a universal Cre recombinase causes recombination of the *Nipbl*^*FLEX*^ allele (at LoxP and lox5171 recognition sites), resulting in progeny carrying the *Nipbl*^*Flrt*^ allele. *Nipbl*^*Flrt*/+^mice are phenotypically wildtype: Image is of male *Nipbl*^*Flrt/+*^ and wildtype littermates at 3 wk of age showing no apparent difference in body size. Scatter plot shows weights of 12-wk-old *Nipbl*^*Flrt/+*^ mice (red, *n* = 19: 6 female; 13 male) and wildtype littermates (black, *n* = 10: 3 female; 7 male) from 3 litters. Expression of *β-geo* was not detected by X-gal staining in E10.5 *Nipbl*^*Flrt/+*^ embryos. qRT-PCR results show relative *Nipbl* expression in kidneys of E17.5 *Nipbl*^*Flrt/+*^ (*n* = 6) compared to wildtype littermates (*n* = 6), plotted as in B; *p* > 0.05 by Student’s *t* test. E. Cre-mediated recombination of mice carrying the *Nipbl*^*Flox*^ allele, obtained by crossing *Nipbl*^*Flox/+*^ mice with *Nanog-Cre* hemizygous mice, results in re-inversion of the SA-*βgeo*-pA cassette and re-trapping of *Nipbl* expression. Resulting progeny (*Nipbl*^FIN/+^ mice) are phenotypically mutant, and survive poorly, with only 13 *Nipbl*^FIN/+^ mice (4%) surviving to weaning age out of 315 total pups born (significantly less than the expected 25% survival, *p* < 0.001 by Chi-square analysis). Adult *Nipbl*^*FIN/+*^ mice are smaller than wildtype littermates: Image is of 6-wk old males; scatter plot shows weights of 8-wk-old *Nipbl*^*FIN/+*^ mice (red, *n* = 11: 4 females; 7 males) compared to wildtype littermates (black, *n* = 7: 3 females; 4 males) from 16 litters. Ubiquitous expression of *β-geo* is detected by X-gal staining. qRT-PCR results show reduced *Nipbl* expression in brains of E17.5 *Nipbl*^*FIN/+*^ (*n* = 7) compared to wildtype littermates (*n* = 6), plotted as in B; asterisk: *p* < 0.05, Student’s *t* test. Scale bars = 1 mm for all panels. Frt (purple triangles), F3 (green triangles), loxP (orange triangles) and lox5171 (yellow triangles); SA, splice acceptor; *β-geo*, *β*-galactosidase/neomycin phosphotransferase fusion gene; pA, bovine growth hormone polyadenylation sequence.

To make it possible to investigate the roles of different cell lineages in the development of heart defects in *Nipbl*-deficient mice, we developed a *Nipbl* allelic series based on embryonic stem (ES) cells bearing a “conditional-invertible” (FLEX, or Flip-Excision [33,53,54]) gene trap in the *Nipbl* locus. We tested several *Nipbl*-gene-trapped ES cells that are available through public repositories, and ultimately selected, verified, and generated mice using ES cells bearing the *Nipbl*^*Gt(EUCE313f02)Hmgu*^ allele (MGI: 4374347, hereafter known as *Nipbl*^*FLEX*^), which is depicted in Fig 2A (see also S2 Fig). The gene-trap vector in these cells is inserted into intron 1 (14.5 kb downstream of exon 1) of the *Nipbl* gene, the same intron in which the gene-trap vector was inserted in the ES cells we used previously to generate *Nipbl*^*+/-*^ mice [22].

This vector introduces a FLEX cassette, which contains a *β-geo* reporter that reports on successful trapping, as well as flanking heterotypic recombinase target sites for both Cre and Flp recombinases [33,53,54], oriented so that exposure to either of these recombinases should lead to irreversible inversion of the trapping vector (due to excision of the cognate binding sites), loss of trapping, and loss of *β-geo* expression. Subsequent exposure to the other recombinase can then be used to induce a second round of irreversible inversion, restoring both trapping and *β-geo* expression. The different configurations of alleles in the *Nipbl*^*FLEX*^ series, as well as the phenotypes of the different mouse lines obtained by successive rounds of recombination, are detailed in Fig 2B–2E, S2 and S3 Figs.

Fig 2B illustrates salient features of *Nipbl*^*FLEX/+*^mice, which were generated directly from chimeras produced using ES cells carrying the *Nipbl*^*FLEX*^ allele. *Nipbl*^*FLEX/+*^ mice are, in accordance with predictions for the first (trapped) allele in the series, haploinsufficient for *Nipbl* and phenotypically similar to *Nipbl*^*+/-*^ mice by every measure tested: small body size, ubiquitous expression of the *β-geo* reporter, and reduced expression of *Nipbl* (assessed by qRT-PCR) (Fig 2B). In addition, *Nipbl*^*FLEX/+*^ mice have a low survival rate, with only about 4% of pups surviving to weaning (4 *Nipbl*^*FLEX/+*^ survivors versus 104 wildtype littermate survivors across 17 litters; S3 Fig). As discussed in the next section, *Nipbl*^*FLEX/+*^ mice also faithfully replicate the heart defects seen in *Nipbl*^*+/-*^ mice.

When *Nipbl* ^*FLEX/+*^ mice are crossed with transgenic *Actin-FlpE* (Fig 2C) or *Nanog-Cre* mice (Fig 2D), to produce the genotypes we designate as *Nipbl*^*Flox/+*^ and *Nipbl*^*Flrt/+*^, respectively, the progeny are normal in phenotype: embryos no longer express *β-geo* (note lack of X-gal staining), *Nipbl* transcript levels are restored to normal, and animals are indistinguishable from wildtype littermates in terms of size, rates of survival, and weight (Fig 2C and 2D and S3 Fig). Finally, when *Nipbl*^*Flox/+*^ mice are crossed with *Nanog-Cre* mice to re-invert the gene-trap and generate what we refer to as *Nipbl*^*FIN/+*^ mice, the *Nipbl*^*FIN/+*^ progeny are again *Nipbl*-deficient, small in size, show ubiquitous *β-geo* expression, and survive poorly (Fig 2E legend). These results demonstrate that mice from the *Nipbl*^*FLEX*^ allelic series can be successfully “toggled” between mutant and wildtype genotypes and phenotypes.

### *Nipbl* ^*FLEX/+*^ and *Nipbl*^*+/-*^ Mice Develop Similar Heart Defects

To confirm that *Nipbl* ^*FLEX*^ mice phenocopy *Nipbl*-deficient mice, we compared a large number of hearts from *Nipbl*^*FLEX/+*^ and *Nipbl*^*+/-*^ embryos (and their wildtype littermates) at E17.5. We used high-resolution (50 μm voxel diameter) magnetic resonance imaging (MRI) to scan rapidly through many specimens. Although this procedure detected heart defects with high accuracy (see Materials and Methods), we frequently confirmed defects by paraffin histology.

**Fig 3:**
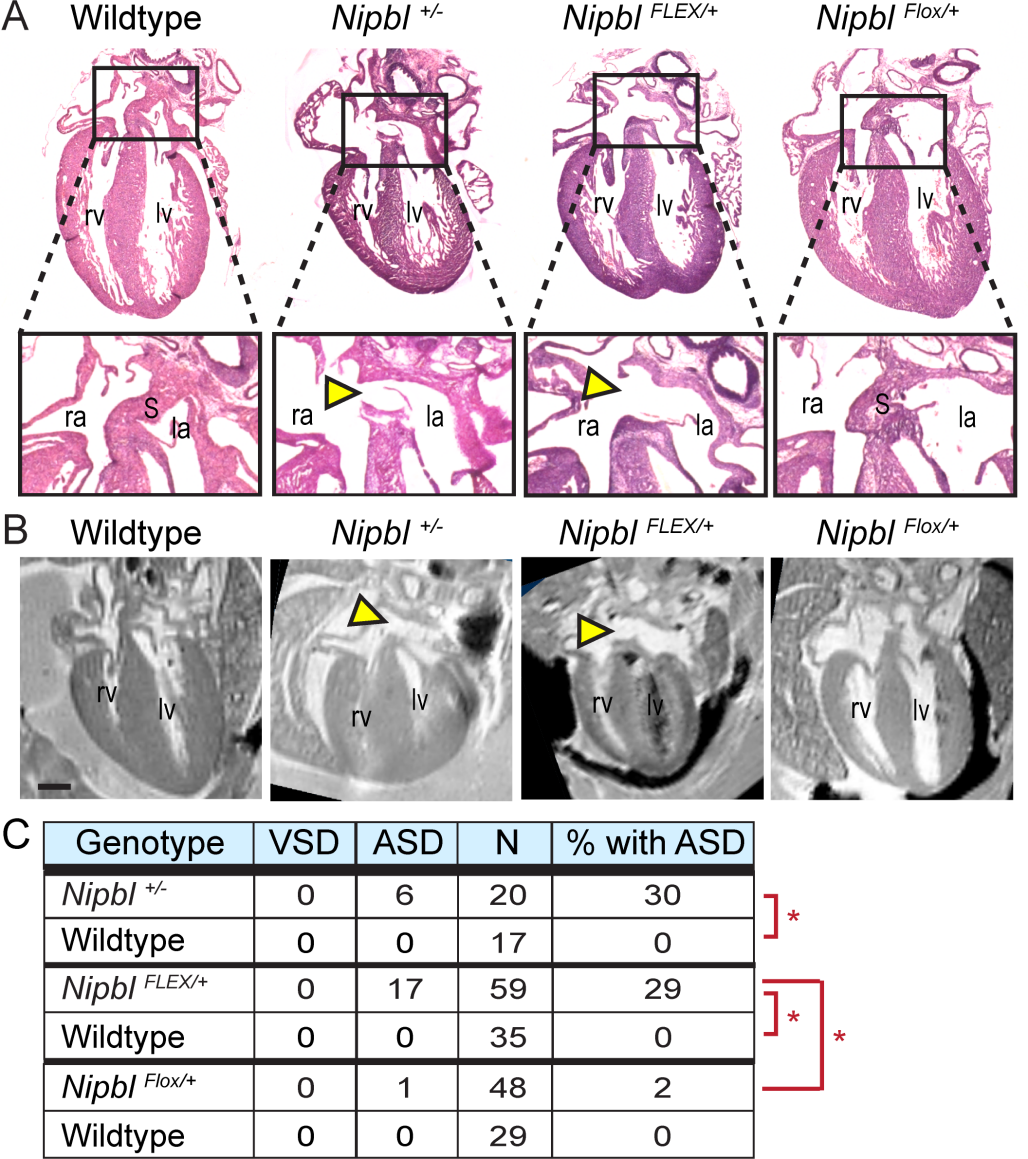
*Nipbl* ^*FLEX/+*^ and *Nipbl*^*+/-*^ mice develop heart defects at the same high frequency. A, B. Paraffin-sectioned hearts stained with H&E (A) and MRI-scanned hearts (B) show large atrial septal defects (yellow arrowheads) in *Nipbl*^*+/-*^ and *Nipbl*^*FLEX/+*^ mice, but not in wildtype or *Nipbl*^*Flox/+*^ mice. Scans and histology were performed on fixed tissue from E17.5 embryos. Scale bar = 500 μm. la, left atrium; lv left ventricle; ra, right atrium; rv right ventricle; S, septum. C. Summary table showing incidence of atrial septal defects (ASDs) and ventricular septal defects (VSDs) in hearts of *Nipbl*^*+/-*^, *Nipbl*^*FLEX/+*^, *Nipbl*^*Flox/+*^ mice and wildtype littermate embryos at E17.5. Asterisks: *p* < 0.01 by Chi-square analysis. Data were pooled from analyses of multiple crosses (see S1 Data: Sample Numbers) and progeny are on various backgrounds depending on parental backgrounds: *Nipbl*^*+/-*^, CD-1; *Nipbl*^*FLEX/+*^, mixed; *Nipbl*^*Flox/+*^, C57Bl6/J or mixed.

The results are shown in Fig 3. At E17.5, both *Nipbl*^*FLEX/+*^ and *Nipbl*^*+/-*^ hearts display large atrial-septal defects (ASDs) at a similar frequency, about 30% (Fig 3). This number is somewhat smaller than previously reported for *Nipbl*^*+/-*^ mice (~50%), because a later time of assessment and more stringent criteria were used here; by these criteria we observed no ASD in wildtype littermates of *Nipbl*^*FLEX/+*^ and *Nipbl*^*+/-*^ mice. We also examined a large number of *Nipbl*^*Flox/+*^ mice, and found only a single ASD among 48 hearts examined (i.e., 2%, Fig 3C). All ASDs observed in *Nipbl*-deficient mice were of the ostium secundum type, similar to what is observed in individuals with CdLS, when ASD is seen as an isolated cardiac defect [15]. Ventricular septal defects were not observed in this analysis, nor were arterial stenoses or obvious abnormalities of ventricular wall thickness (S4 Fig). We did note that the hearts of *Nipbl*-deficient mice, whether *Nipbl*^*FLEX/+*^ or *Nipbl*^*+/-*^, are noticeably smaller than those of wildtype littermates—to about the same degree that *Nipbl*-deficient embryos themselves are smaller than wildtypes.

### Characterization of Cre Lines for Tissue-Specific Manipulation of *Nipbl* Deficiency

In principle, mice bearing *Nipbl*^*FLEX*^ and *Nipbl*^*Flox*^ alleles (Fig 2B and 2C) can be used to determine in which cells or tissues *Nipbl*-deficiency is either necessary or sufficient to cause the heart defects that arise in globally *Nipbl*-deficient embryos. We selected six different Cre-expressing mouse lines to use in such experiments. Two of them—*Nkx2-5-*Cre [55] and *cTnt-*Cre [56]—express Cre in the cardiac crescent and developing cardiomyocytes. Two others, *Sox17-A2-iCre* [57] and *FoxA2-2A-iCre* [58] have been reported to express Cre primarily in early endoderm, at a time when cardiac progenitors receive important developmental signals from this tissue [31]. *Wnt1*-*Cre* [59] was chosen because it expresses Cre in the neural crest and its derivatives, including portions of developing heart and outflow tracts [34]. Finally, *Nanog*-*Cre* served as a positive control, as it expresses Cre in all cells of the developing embryo [60].

**Fig 4:**
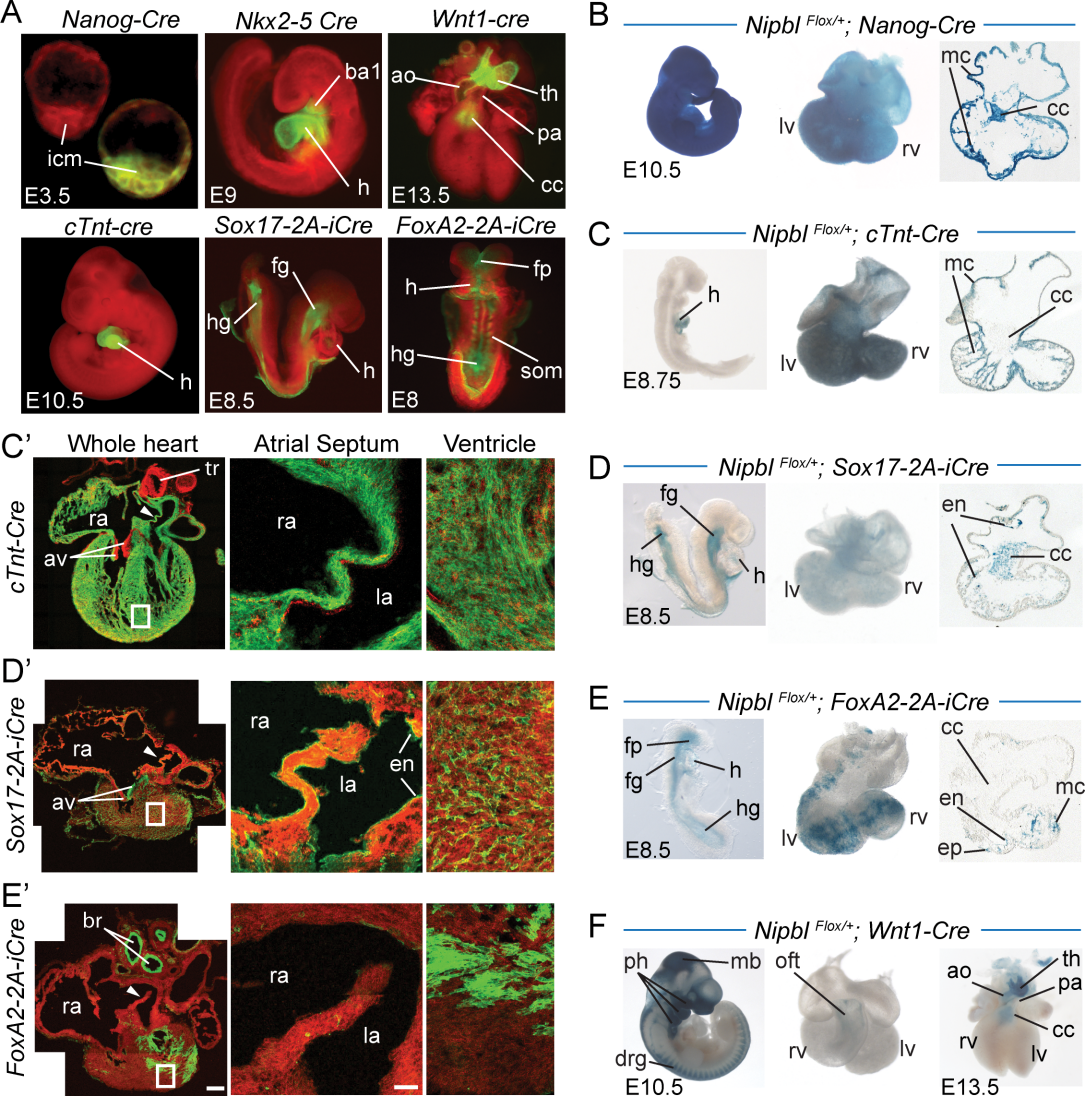
Domains of Cre expression in mice made *Nipbl*-deficient in different heart developmental lineages. A. Relevant patterns of Cre expression from the six Cre lines used in this study (*Nanog-Cre*, *Nkx2-5-Cre*, *cTnt-Cre*, *Sox17-2A-iCre*, *FoxA2-2A-iCre*, *Wnt1-Cre*), assessed on the *Td-Tomato-EGFP* reporter during early embryogenesis. Cre-mediated recombination is indicated by expression of EGFP. Note two embryos are shown in the *Nanog-Cre* panel; only the embryo on the right carries the *Nanog-Cre* allele and exhibits EGFP expression in the inner cell mass (icm). B–F. X-gal staining was performed to detect expression of *β-geo* in *Nipbl*^*Flox/+*^ embryos carrying one of the Cre transgenes. X-gal staining was assessed in whole embryos (at E8–10.5, left panels), whole hearts (at E10.5, middle panels in B-E and at E13.5 in *Nipbl*^*Flox/+*^*;Wnt1-cre* hearts, right panel in F), and sectioned hearts (at E10.5, right panels B-E). C’–E’. Confocal images of E15.5 hearts generated from crosses of *Nipbl*^*Flox/+*^;*Td-Tomato-EGFP* mice with different Cre-expressing lines. (C’) *cTnt-Cre*: EGFP in myocardium including atrial septum. (D’) *Sox17-2A-iCre*: EGFP in endocardium, and small blood vessels. (E’) *FoxA2-2A-iCre*: EGFP in both endoderm (bronchi linings) and some mesoderm (muscle in ventricle); minimal EGFP expression in atrial septum (note that pattern is different from *Sox17-2A-iCre*). ao, aorta; av, atrioventricular valve; ba1, branchial arch; br, bronchi; cc, cardiac cushion; drg, dorsal root ganglion; en, endocardium; ep, epicardium; fp, floor plate; fg, foregut; h, heart; hg, hindgut; icm, inner cell mass; la, left atria; lv, left ventricle; mb, midbrain; mc, myocardium; oft, outflow tract; pa, pulmonary artery; ph, pharyngeal arches; ra, right atria; rv, right ventricle; som, somites; th, thymus; tr, trachea

To verify the domains of Cre expression in these lines, we crossed them with *Td-tomato-EGFP* reporter mice [61], which express a membrane-targeted tomato red fluorescent protein that is replaced by a membrane-targeted enhanced green fluorescent protein (EGFP) when Cre-mediated excision occurs. As shown in Fig 4A, *Nanog-Cre* embryos express EGFP in all cells of the blastocyst inner cell mass, as expected. *Nkx2-5-Cre* and *cTnt-Cre* embryos express EGFP primarily in heart at E9–10.5, with some EGFP apparent in the first branchial arch for *Nkx2-5-Cre*. At E13.5, hearts of *Wnt1-Cre* embryos express EGFP in the inner lining of the great arteries and cardiac cushion, consistent with the distribution of neural crest contributions to the heart [62]. In *Sox17-2A-iCre* and *FoxA2-2A-iCre* embryos at E8–8.5, EGFP expression is observed in endodermal derivatives (foregut and hindgut), with small numbers of EGFP+ cells in developing heart. *FoxA2-2A-iCre* embryos also displayed some ectodermal (floorplate) and mesodermal (somite) EGFP.

Because recombination of the *Nipbl*^*Flox*^ allele reactivates expression of *β-geo*, we could also use X-gal staining to document patterns of *Nipbl* inactivation produced by these Cre lines (Fig 4B–4F). These results confirm that *Nanog-Cre* drives ubiquitous recombination (Fig 4B), while *cTnt-Cre* drives recombination specifically in the heart, and solely in myocardium (note sparing of the cardiac cushion in Fig 4C). *Sox17-2A-iCre* drives recombination in endodermal derivatives such as foregut and hindgut at E8.5, with additional staining in the heart that, by E10.5, can be seen to correspond to endocardium and cardiac cushion (Fig 4D), a pattern complementary to *cTnt-Cre*. Although *FoxA2* is often regarded as an endodermal marker, the pattern of recombination driven by *FoxA2-2A-iCre* is more complex (consistent with its broader expression pattern compared to *Sox17-2A-iCre* in Fig 4A), and eventually comes to include sub-portions of epi-, myo-, and endocardium, while mainly sparing the anterior heart, outflow tracts and cardiac cushion (Fig 4E). *Wnt1-Cre* embryos show essentially no recombination in the heart proper at E10.5, with X-gal staining appearing by E13.5 in a pattern confined to the cardiac cushion and developing great arteries (Fig 4F).

These results document that these Cre lines can be used to manipulate *Nipbl* expression in essentially all of the major tissues that contribute to, or influence the development of, the heart. Furthermore, while some show overlapping patterns of recombination, *cTnt-Cre* and *Sox17-2A-iCre* are essentially complementary to one another—the former acting within the cardiomyocyte lineage, and the latter acting within endoderm plus non-cardiomyocyte mesodermal derivatives within the heart: the endocardium, endocardially-derived cells of the cardiac cushion, and vascular endothelium (these correspond to known domains of *Sox17* expression [63]). Indeed, high magnification views of *Td-tomato-EGFP* reporter expression at E15.5 show that *cTnt-Cre* (Fig 4C’) recombines in the vast majority of heart cells, including cells of the atrial septum, but spares endocardium, the cushion-derived atrioventricular valves, and scattered EGFP-negative cells in the ventricular parenchyma (most likely endothelial cells lining small blood vessels). In contrast, *Sox17-2A-iCre* spares the myocardium, but drives recombination in endocardium, atrioventricular valves, and small blood vessels throughout the ventricles (Fig 4D’).

### Effects of *Nipbl* Deficiency in Single Developmental Lineages

**Fig 5:**
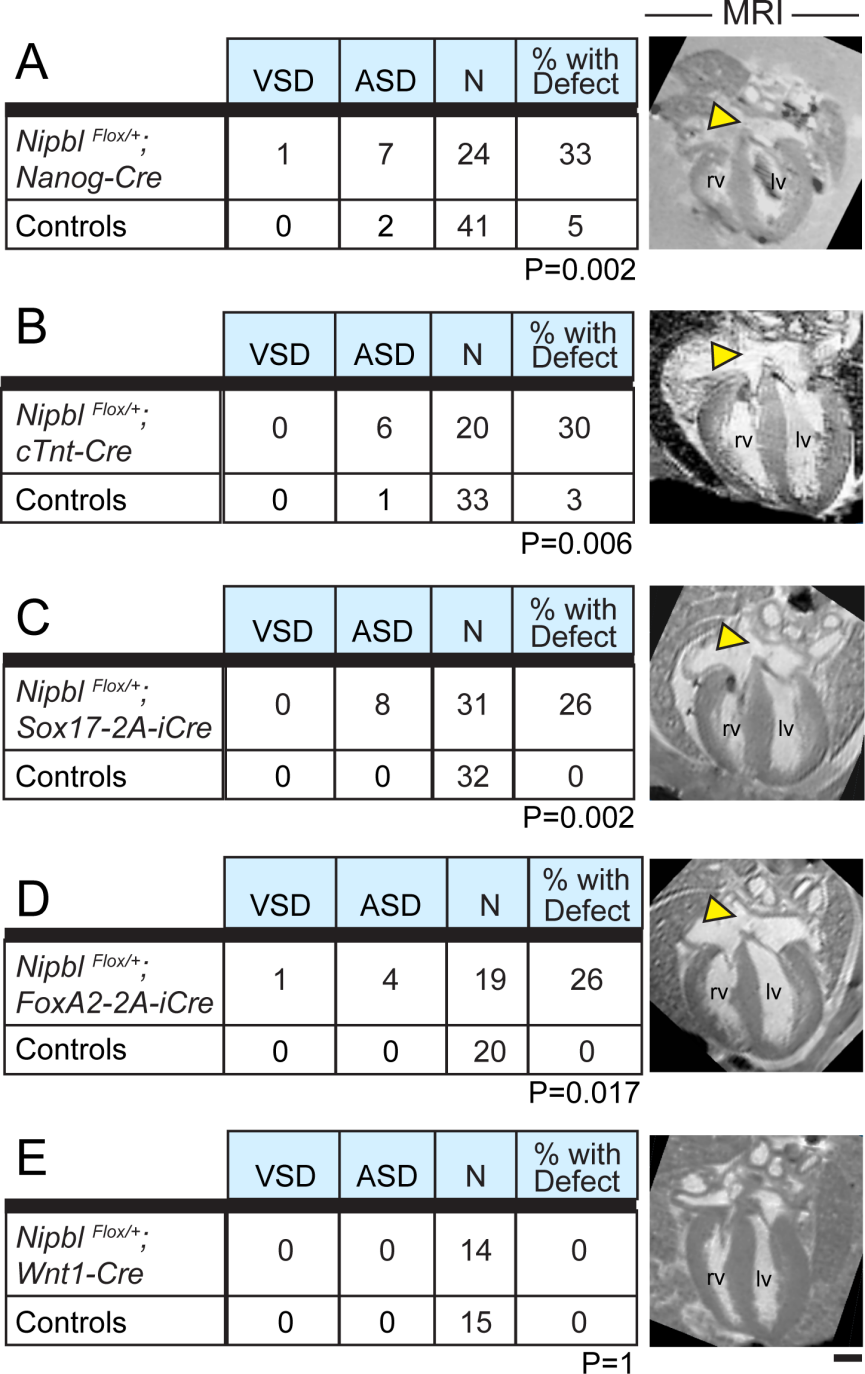
Creation of *Nipbl* deficiency in cardiac developmental lineages. *Nipbl*^*Flox/+*^ mice were crossed with mice hemizygous for each of five indicated Cre-expressing transgenes. MRI analysis of hearts was performed at E17.5. A–D show that whether *Nipbl* was made deficient in all tissues (*Nanog-Cre*, A), specifically in cardiomyocytes (*cTnt-Cre*, B), or primarily in endoderm-derived tissues (*Sox17-2A-iCre*, C) or mixed cardiac lineages (*FoxA2-2A-iCre*, D), the incidence of CHDs (primarily ASDs) was approximately 30%. (Chi-square analyses indicate that frequencies of heart defects observed in embryos with *Nipbl* deficiency in experiments A–D do not differ significantly from each other [*p* > 0.4 for each pairwise comparison].) In contrast, control hearts (a mix of wildtype, *Nipbl*^*Flox/+*^, and *Cre*+ littermates for each specific cross; see S1 Data: Sample Numbers) had an incidence of heart defects ranging from 0%–5% (A–D). *Nipbl* deficiency in the *Wnt1* domain (neural crest) did not give rise to heart defects (E). CHDs occurred primarily in the form of ASDs of the ostium secundum type (yellow arrowheads); VSDs observed in A and D are of the perimembranous type. *p*-Values are from Chi-square analyses and are indicated for each corresponding cross. Scale bar = 500 μm. lv, left ventricle; rv, right ventricle.

We first crossed mice carrying *Cre*-expressing transgenes with *Nipbl*^*Flox/+*^ mice, to yield progeny in which *Nipbl* deficiency is introduced into specific lineages, while the rest of the embryo retains normal *Nipbl* expression (Fig 5). *Nanog*-*Cre* was used as a control to produce mice that were *Nipbl*-deficient in all tissues. When *Nipbl*^*Flox/+*^;*Nanog*-*Cre* progeny were analyzed at E17.5, 33% of hearts displayed heart defects, the vast majority of which were large ASDs (Fig 5A). These results are similar to those observed for E17.5 hearts from both *Nipbl*^*+/-*^ and *Nipbl*^*FLEX/+*^ mice (Fig 3).

Next, *cTnt-Cre* was used to create *Nipbl* deficiency specifically in cardiomyocytes (Fig 5B). In this case, large ASDs were also found at a frequency of 30%, not significantly different from that seen with *Nipbl*^*+/-*^, *Nipbl*^*FLEX/+*^, or *Nipbl*^*Flox/+*^;*Nanog-Cre* progeny (Figs 3C and 5A, S1 Data). This finding suggested that *Nipbl* deficiency in cardiomyocytes accounts for the high incidence of heart defects seen in globally *Nipbl*-deficient animals.

However, subsequent experiments suggested otherwise. Use of either *Sox17-2A-iCre* (expressed in endoderm and non-cardiomyocyte mesodermal derivatives) or *FoxA2-2A-iCre* (expressed both in endodermal and multiple other derivatives) to create *Nipbl* deficiency also resulted in a high incidence of ASD, about 26% in each case (Fig 5C and 5D). This is nearly as high as, and not statistically significantly different from, the level caused by either global or cardiomyocyte-specific *Nipbl*-deficiency (S1 Data).

Finally, we used *Wnt1-Cre* to investigate the role of neural crest. The neural crest not only contributes cells to cardiac structures, such as outflow tracts and valves, migrating neural crest cells interact in important ways with other cells that contribute to the heart [31,64]. Smith et al. [65] have suggested that *Nipbl* deficiency in mice impairs the functioning of cranial neural crest, and Schuster et al., [66], using zebrafish, recently proposed a neural crest origin for heart defects in cohesinopathies. In our experiments, however, no heart defects were seen in *Nipbl*^*Flox/+*^;*Wnt1-Cre* progeny (Fig 5E), indicating that *Nipbl* deficiency in the neural crest, at least on its own, is not sufficient to produce heart defects.

Overall our results show that when cells derived from cardiogenic mesoderm, endoderm, or subpopulations of both are made deficient in *Nipbl*, heart defects, primarily ASD, always develop at a frequency of approximately 30%, the same incidence as observed in mice that are globally *Nipbl*-deficient (Figs 3 and 5A, and S1 Data). This is a striking finding, since it suggests that the effects of *Nipbl*-deficiency in different cardiac developmental lineages, even non-overlapping lineages, are not additive.

### Restoration of *Nipbl* Expression to Either of Two Distinct Lineages Rescues Heart Defects

In experiments complementary to those described above, we crossed *Nipbl*^*FLEX/+*^ mice with Cre-expressing transgenic mice, to produce embryos that are globally *Nipbl*-deficient except within those lineages in which Cre recombinase acts. We focused on three Cre-expressing lines: *Nanog-Cre*, *cTnt-Cre*, and *Sox17-2A-iCre*.

**Fig 6:**
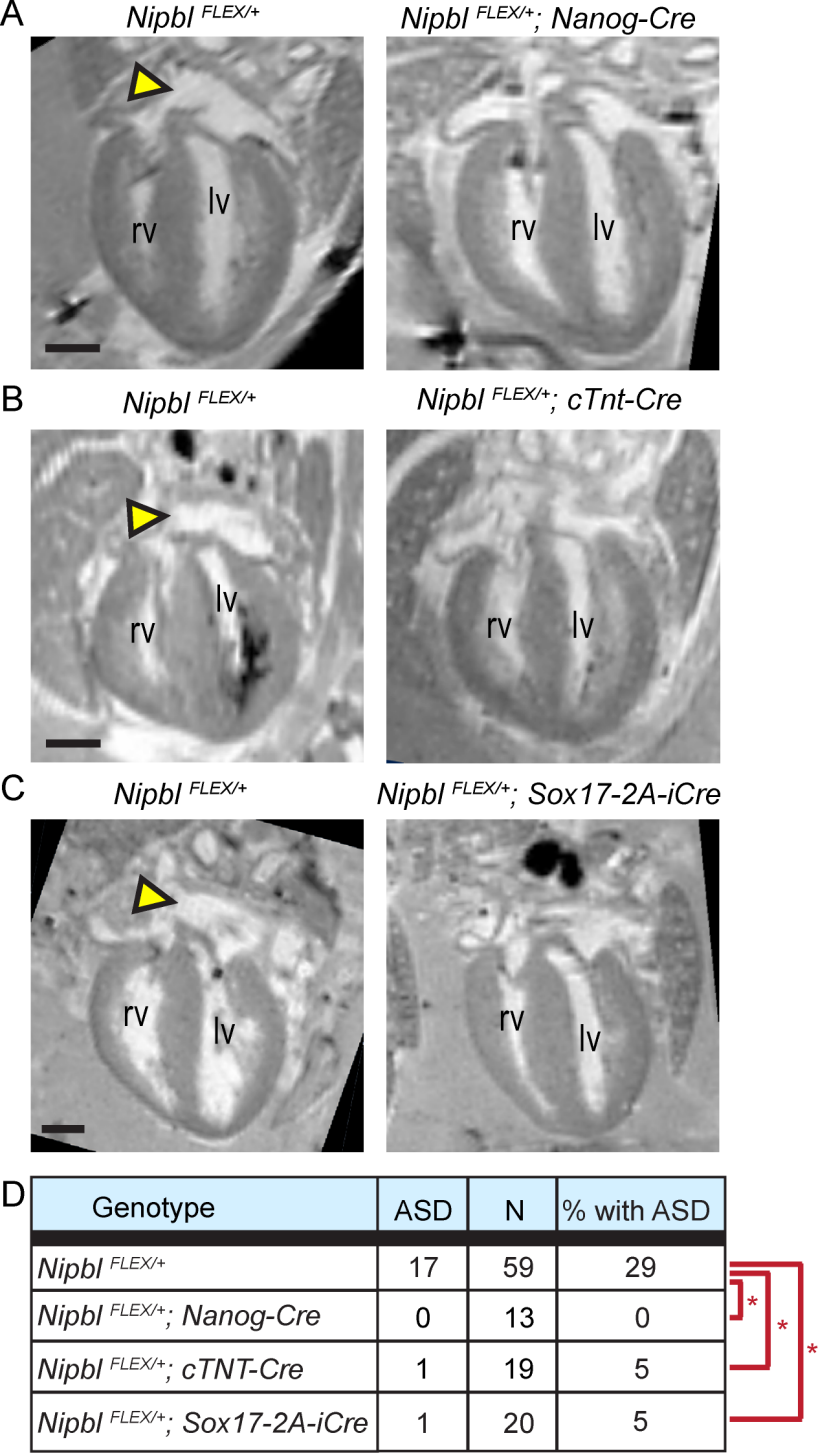
Restoration of *Nipbl* expression in different heart lineages rescues heart defects. MRI analysis of E17.5 hearts show that *Nipbl*^*FLEX/+*^ mice exhibit ASDs at a frequency of ~30% (yellow arrowheads in A–C, and D). The incidence of heart defects was significantly reduced in mice in which *Nipbl* was restored in all tissues (*Nipbl*^*FLEX/+*^*;Nanog-Cre*), specifically in the *cTnt* domain (*Nipbl*^*FLEX/+*^*;cTnt-Cre*), or specifically in the *Sox17* domain (*Nipbl*^*FLEX/+*^*;Sox17-2A-iCre*) (D, asterisks: Chi-square, *p* < 0.05). Chi-square analysis on the incidence of heart defects between the three rescued lines indicated no significant difference from one another (Chi-square, *p* > 0.71). Progeny are on various backgrounds depending on parental backgrounds: *Nipbl*^*FLEX/+*^ (mixed), wildtype (CD-1), *cTnt-Cre* (CD-1), *Nanog-Cre* (C57Bl6/J), and *Sox17-2A-iCre* (C57Bl6/J); the incidence of heart defects in *Nipbl*^*FLEX/+*^ embryos from each of these crosses did not differ significantly (*p* > 0.89 by Chi-square analysis; S1 Data). Size bar = 500 μm. lv, left ventricle; rv, right ventricle

As shown in Fig 6A, the hearts of *Nipbl*^*FLEX/+*^;*Nanog-Cre* mice lacked ASDs, and were phenotypically indistinguishable from wildtype, as expected for a global rescue of *Nipbl* expression. Interestingly, in *Nipbl*^*FLEX/+*^;*cTnt-Cre* hearts, the incidence of ASDs was also very low: of 19 hearts analyzed, only 1 displayed an ASD (~5%), which is significantly different from *Nipbl*^*FLEX/+*^, and statistically indistinguishable from wildtype (S1 Data). Remarkably, *Nipbl*^*FLEX/+*^;*Sox17-2A-iCre* hearts also displayed a very low incidence of ASDs (5%, Fig 6C and 6D) that was indistinguishable from wildtype (S1 Data). Chi-square analysis indicated that, for all three types of *Nipbl*^*FLEX/+*^;*Cre* embryos, none was distinguishable from any other in terms of the observed incidence of heart defects, but all were significantly different from *Nipbl*^*FLEX/+*^ (Fig 6D).

### Key Determinants of ASD Risk Most Likely Lie Elsewhere in the Embryo

The above results indicate that *Nipbl* deficiency in *either* of two non-overlapping sets of cells—the “*cTnt* lineage”, by which we mean descendants of *cTnt*-*Cre* expressing cells, and the “*Sox17* lineage”, by which we mean descendants of *Sox17*-*Cre* expressing cells)—will cause heart defects, while rescue of *Nipbl* deficiency in either of the same two populations rescues those defects. With respect to the creation of heart defects, the first result implies that *Nipbl* deficiency in *either* lineage is sufficient, while the second result implies that *Nipbl* deficiency in *neither* lineage is sufficient—which certainly seems paradoxical.

The problem is highlighted in tabular form in Table 1. *Nipbl*^*Flox/+*^;*Cre* experiments (“conditional haploinsufficiency,” Fig 5) and *Nipbl*^*FLEX/+*^;*Cre* experiments (“conditional rescue,” Fig 6) both generate embryos in which cardiomyocytes are *Nipbl*-deficient, and endoderm, endocardium, and vascular endothelium are not; or endoderm, endocardium, and vascular endothelium are deficient and cardiomyocytes are not. Yet opposite results, with respect to heart defects, are obtained for the same genotypes in the two types of experiments. The key to resolving this apparent paradox is to remember the additional variable that distinguishes the two classes of experiments: the rest of the embryo. In conditional haploinsufficiency experiments, all lineages outside the one in which Cre acts are wildtype. In conditional rescue experiments, all lineages outside the one in which Cre acts are *Nipbl* deficient.

**Table 1:** The incidence of atrial septal defects (ASD) as a function of *Nipbl* genotype in the heart.

Conditional haploinsufficiency experiments (*Flox*/+ x Cre)		
*Nipbl* genotype in cardiomyocyte (*cTnt*) lineage	*Nipbl* genotype in *Sox17* lineage	Frequent ASDs?
+/+	+/+	no
+/-	+/+	yes
+/+	+/-	yes
Conditional rescue experiments (*FLEX*/+ x Cre)		
*Nipbl* genotype in cardiomyocyte (*cTnt*) lineage	*Nipbl* genotype in *Sox17* lineage	Frequent ASDs?
+/+	+/+	no
+/-	+/+	no
+/+	+/-	no

For this difference between the genotypes of the rest of the embryo in the two experimental approaches to explain the results, a critical determinant of ASD risk would have to lie in some lineage other than those represented by *cTnt* and *Sox17*. Furthermore, in that lineage, risk would have to be conferred by being *Nipbl*–wildtype, while protection would have to be conferred by being *Nipbl*-deficient. That would explain why when the rest of the embryo is *Nipbl*–wildtype, ASDs arise when either cardiac lineage is *Nipbl*-deficient, whereas when the rest of the embryo is *Nipbl*-deficient, ASDs arise only when both are.

**Fig 7:**
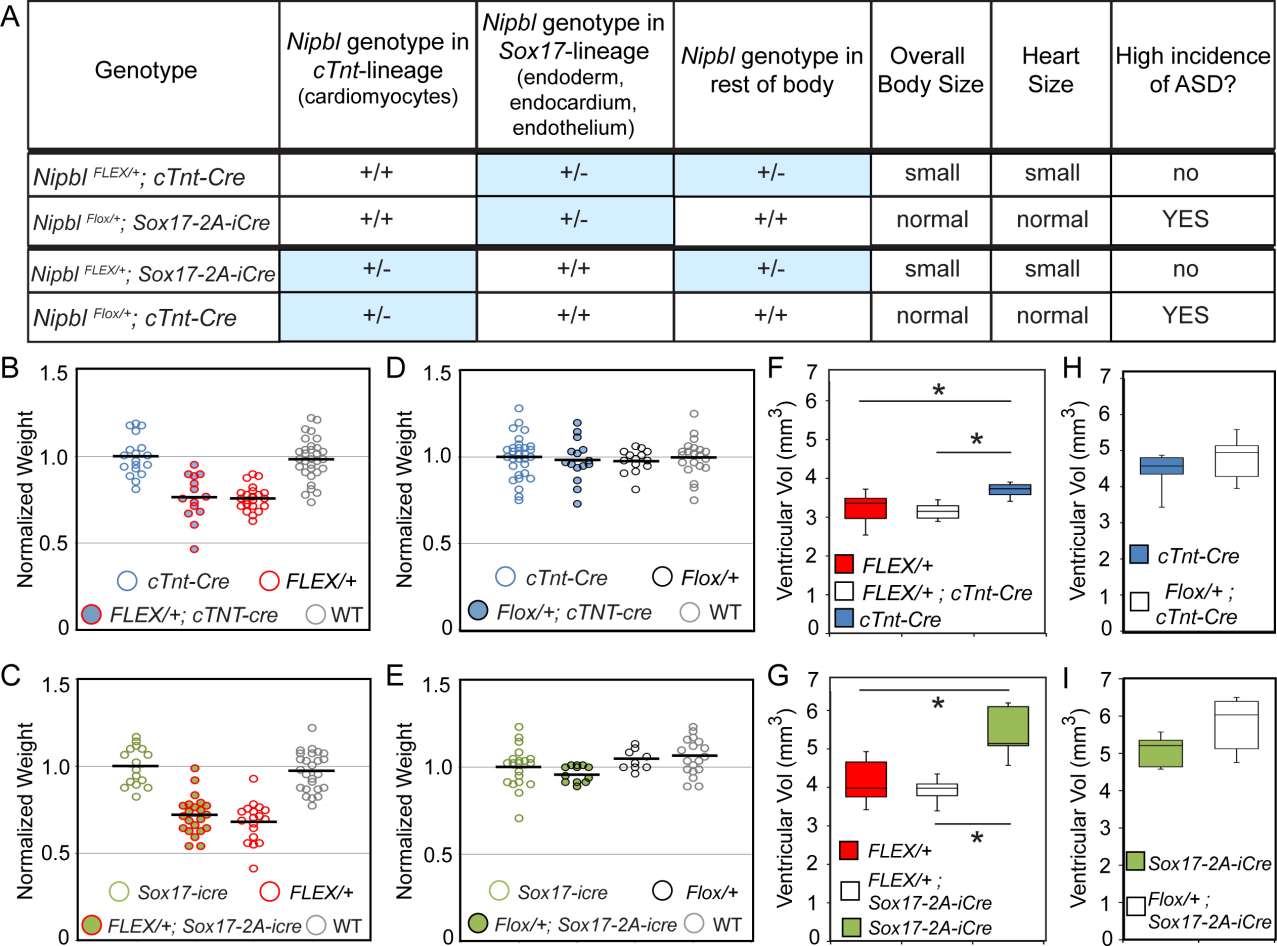
Relationships between *Nipbl* genotype, embryo size, heart size, and ASDs. A. Table summarizing genotypes, heart size, body size and incidence of ASDs in different crosses. B. Rescued *Nipbl*^*FLEX/+*^*;cTnt-Cre* embryos (*n* = 14) resembled their *Nipbl*^*FLEX/+*^ littermates (*n* = 22) in body size and were smaller than control littermates (*cTnt-Cre n* = 18, wildtype *n* = 31). C. Similar results were observed in rescued *Nipbl*^*FLEX/+*^*;Sox17-2A-iCre* embryos (*n* = 22; *Sox17-2A-iCre n* = 16; *Nipbl*^*FLEX/+*^ *n* = 18; wildtype *n* = 25). D. *Nipbl*^*Flox/+*^*;cTnt-Cre* (*n* = 15) were similar in overall body size to littermate controls (*Nipbl*^*Flox/+*^ *n* = 14, *cTnt-Cre n* = 30, wildtype *n* = 21). E. Similar results were observed in *Nipbl*^*Flox/+*^*;Sox17-2A-iCre* (*n* = 13) when compared to littermate controls (*Sox17-2A-iCre*, *n* = 20; *Nipbl*^*Flox/+*^ *n* = 10; wildtype *n* = 19). Note that individual weights for each cross in B–E were normalized to the mean weight of *cTnt-Cre* controls (B, D), or *Sox17-2A-iCre* controls (C, E); black bars indicate normalized mean weight for each genotype. F. Ventricular volume analyses (graphed as box plots) show that the overall heart size of rescued *Nipbl*^*FLEX/+*^*;cTnt-Cre* embryos (*n* = 7) were similar in size to *Nipbl*^*FLEX/+*^ heart size (*n* = 9) (Mann-Whitney U, *p* > 0.05). Control hearts (*cTnt-Cre*, *n* = 6) were significantly larger than the hearts of their *Nipbl*^*FLEX/+*^ and *Nipbl*^*FLEX/+*^*;cTnt-Cre* littermates (asterisks: Mann-Whitney U, *p* < 0.05). G. Rescued *Nipbl*^*FLEX/+*^*;Sox17-2A-iCre* embryo hearts (*n* = 5) were also similar in size to *Nipbl*^*FLEX/+*^ littermate heart size (*n* = 6) (Mann-Whitney U, *p* > 0.05) and significantly smaller than control hearts (*Sox17-2A-iCre*, *n* = 5) (asterisk: Mann-Whitney U, *p* < 0.05). H. Ventricular volume analysis show that the ventricle size of *Nipbl*^*Flox/+*^*;cTnt-Cre* embryos (*n* = 9), which display a high frequency of heart defects, were similar in size to control hearts (*cTnt-Cre*, *n* = 9) (Mann-Whitney U, *p* > 0.05). I. *Nipbl*^*Flox/+*^*;Sox17-2A-iCre* mutant hearts (*n* = 5), which also display a high frequency of heart defects, were also similar in size to control hearts (*Sox17-2A-iCre*, *n* = 5) (Mann-Whitney U, *p* > 0.05).

The possibility that an additional lineage protects against heart defects when *Nipbl*-deficient led us to consider ways in which essentially non-cardiac developmental events might affect the heart indirectly. One of the most penetrant phenotypes of *Nipbl*-deficiency is reduced body size (by ~20% at birth; [22] and S3 Fig, Fig 7 below). Not surprisingly, the determinants of body size lie outside the heart. As shown in Fig 7A–7E, in the *Nipbl*^*Flox/+*^ and *Nipbl*^*FLEX/+*^ crosses described above, body size is determined by *Nipbl*-status outside of the *cTnt* and *Sox17* lineages (i.e., all carriers of the *Nipbl*^*FLEX*^ allele are small, and all carriers of the *Nipbl*^*Flox*^ allele are normal in size). Yet it is also observed that heart size (measured as total ventricular volume) correlates strongly with body size (Fig 7F–7I). Thus, lineages outside the heart and endoderm apparently determine the size of the embryonic heart, just as heart and body size are known to scale together in adults [67]. Below (see Discussion), we raise the possibility that the results in Table 1 might be explained by an influence of heart size on ASD risk, with large hearts being at greater risk for defects than small ones.

### Understanding the Incomplete Penetrance of Heart Defects

One of the puzzling aspects of the above experiments is that the incidence of ASDs always seems to be either very low (≤5%) or about 30%, regardless of genotype. Particularly surprising is the lack of increase in incidence when the entire embryo is *Nipbl*-deficient, as opposed to a single lineage (e.g., Fig 5A–5C). Remarkably, a similar 30% incidence of heart defects is also seen clinically in CdLS [14,15].

We wondered whether this 30% penetrance “ceiling” is a peculiarity of the specific gene expression disturbances caused by *Nipbl* deficiency, or whether it reflects something about the overall state of hearts at the time that *Nipbl*-sensitive defects emerge. For example, is it simply that, at that stage, 30% of hearts are more labile to disturbances overall (e.g., due to embryo-to-embryo variations in the intrauterine environment)?

To explore this idea, we decided to make mice doubly-heterozygous for *Nipbl* and *Nkx2-5*. As discussed previously, *Nkx2-5* is a key, early cardiac transcription factor. Moreover, *Nkx2-5* gene dosage is clearly important in heart development, because haploinsufficiency for *NKX2-5* gives rise to congenital heart disease in man [68,69]. As shown above (Fig 1F), early expression of *Nkx2-5* is reduced in *Nipbl*^*+/-*^ embryos, so it is reasonable to suspect that at least some of the cardiac phenotype of *Nipbl*^*+/-*^ embryos arises from a deficiency in *Nkx2-5*. Combining *Nipbl* and *Nkx2-5* heterozygous mutations should then provide us with an opportunity to observe the effects of lowering *Nkx2-5* levels even further.

For these experiments, we used a knock-in *Nkx2-5*^*Cre*^ allele as a null allele [55,70]. We confirmed that *Nkx2-5*^*Cre/+*^ hearts do in fact express *Nkx2-5* at half the wildtype level using qRT-PCR (S5 Fig), and hereafter refer to them as *Nkx2-5*^*+/-*^ mice (it is the null state of this allele that also made it unsuitable for use in the conditional experiments in Figs 5 and 6). We crossed *Nkx2-5*^*+/-*^ mice with our original *Nipbl*^*+/-*^ mouse line, in which *Nipbl* is not flanked by LoxP sites [22] (so the Cre produced by the *Nkx2-5* transgenic mouse would be irrelevant), and evaluated hearts at E17.5.

**Fig 8:**
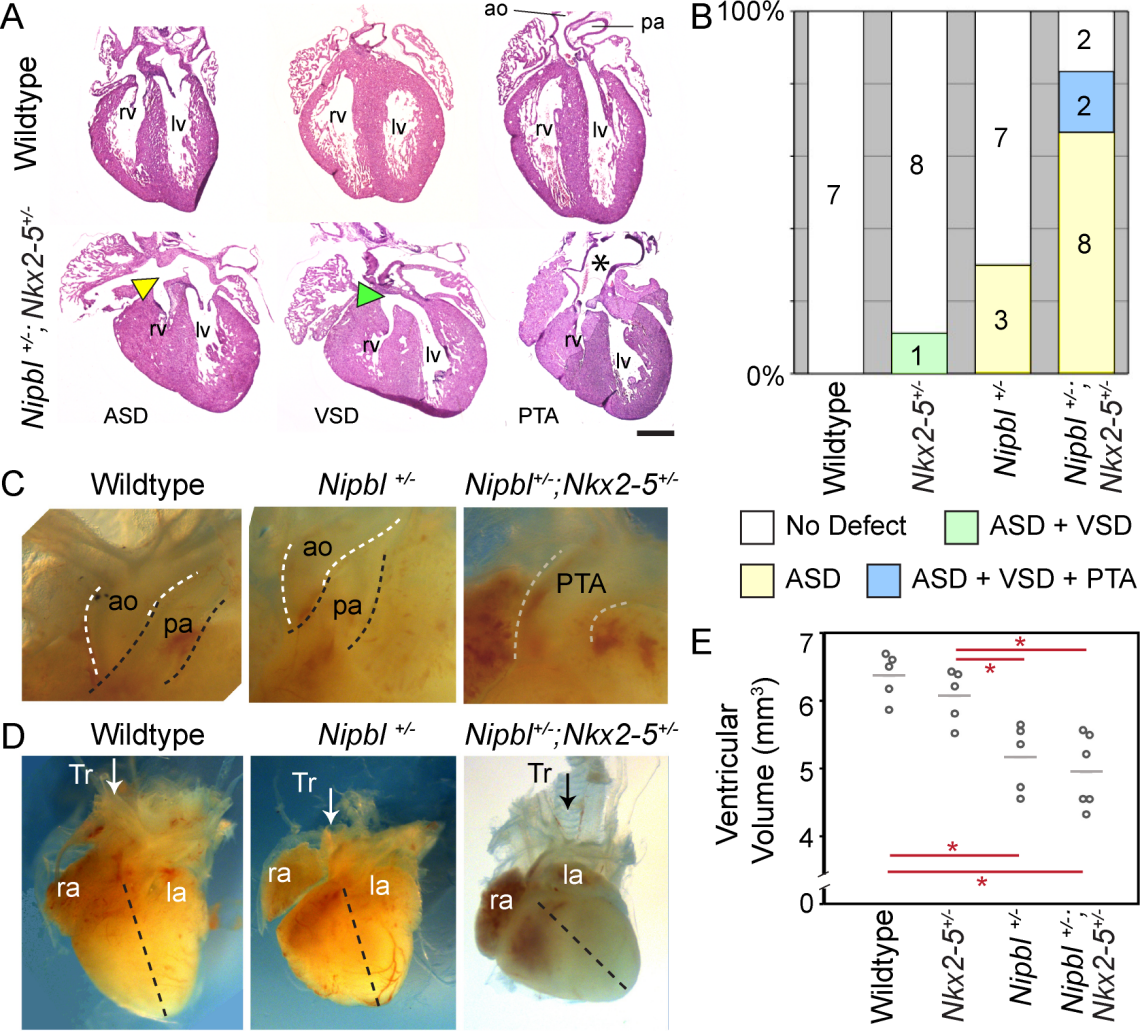
Haploinsufficiency for *Nkx2-5* increases the incidence and severity of heart defects in *Nipbl*-deficient embryos. A. *Nipbl*^*+/-*^*;Nkx2-5*^*+/-*^ mice displayed ASDs (yellow arrowhead), VSDs (green arrowhead), and/or PTAs (black asterisk). B. Histogram showing increases in frequency and types of heart defects in *Nipbl*^*+/-*^*;Nkx2-5*^*+/-*^ hearts compared to littermates of various genotypes; number of hearts observed with each type of defect is indicated. Data show that the overall incidence of defects in *Nipbl*^*+/-*^*;Nkx2-5*^*+/-*^ hearts (83%) is significantly greater than in either *Nipbl*^*+/-*^ (30%, *p* = 0.011 by Chi square) or *Nkx2*.*5*^*+/-*^ (13%, *p* = 0.001 by Chi-square) hearts. C. Gross overview of the great vessels highlighting PTA in *Nipbl*^*+/-*^*;Nkx2-5*^*+/-*^ mice. D. Heart position and morphology in a subset of *Nipbl*^*+/-*^*;Nkx2-5*^*+/-*^ mice was drastically different than in littermates. Arrows indicate the position of the trachea (Tr), dotted lines originate at the pulmonary artery and ends at the apex of the heart; note drastic change in the angle of dotted line in *Nipbl*^*+/-*^*;Nkx2-5*^*+/-*^ heart. E. Ventricular volumes for hearts of each genotype; horizontal bars indicate means. Ventricular volumes of *Nipbl*^*+/-*^*;Nkx2-5*^*+/-*^ hearts (*n* = 6) were similar in size to *Nipbl*^*+/-*^ hearts (*n* = 5) (Mann-Whitney U, *p* > 0.05), whereas control hearts (wildtype *n* = 5 and *Nkx2-5*^*+/-*^, *n* = 5) were significantly larger than the hearts of their *Nipbl*^*+/-*^ and *Nipbl*^*+/-*^*;Nkx2-5*^*+/-*^ littermates (red asterisks: Mann-Whitney U, *p* < 0.05). ao, aorta; ASD, atrial septal defect; la, left atrium; lv, left ventricle; pa, pulmonary artery; PTA, persistent truncus arteriosus; ra, right atrium; rv, right ventricle; Tr, trachea; VSD, ventricular septal defect; Size bar = 500 μm.

As shown in Fig 8, even though *Nkx2-5*^*+/-*^ mice only rarely display heart defects on their own, *Nipbl*^*+/-*^; *Nkx2-5*^*+/-*^ mice exhibit a much higher incidence of heart defects than *Nipbl*^*+/-*^ hearts (83% versus 30%), and a spectrum of defects that is both more varied in type and more severe (Fig 8A and 8B). VSDs as well as ASDs were seen in *Nipbl*^*+/-*^; *Nkx2-5*^*+/-*^ hearts, in several cases in combination; and two cases of persistent truncus arteriosus (PTA) were also observed (Fig 8A–8C). Also apparent was a change in the angle of a subset of these hearts (Fig 8D), suggestive of malrotation during development. Otherwise, *Nipbl*^*+/-*^;*Nkx2-5*^*+/-*^ hearts are similar in size (Fig 8E) and histology to *Nipbl*^*+/-*^ hearts.

These results indicate that haploinsufficiency for *Nkx2-5* can markedly enhance both the incidence and severity of defects caused by *Nipbl-*deficiency, well above the 30% seen in experiments in which lineages that display *Nipbl*-deficiency were individually manipulated. Overall, the results imply that such heart defects are inherently sensitive to quantitative modulation in the majority of embryos. Thus, the lack of significant phenotypic difference among hearts in conditional *Nipbl* haploinsufficiency experiments in Fig 5A–5D, requires an alternative explanation (see Discussion).

## Discussion

The *Nipbl*^+/-^ mouse—a model of Cornelia de Lange Syndrome (CdLS)—provides a unique resource for studying how the combinatorial effects of genetic variation cause birth defects, because the sole consequence of *Nipbl* haploinsufficiency appears to be small quantitative adjustments to the levels of expression of hundreds to a thousand genes [21–23,25]. As described above, *Nipbl*^+/-^ mice display large ASDs—one of the most common CHDs in the general population—at a frequency of about 30%, similar to the incidence of CHD in CdLS.

Not only do *Nipbl*^+/-^ mice display defects of the atrial septum, which normally forms and closes by E14.5 [30], they exhibit delayed closure of the ventricular septum, which normally takes place a day earlier (Fig 1A), and reduction in the size of the right ventricle several days before that (Fig 1C and 1D). Even earlier, at the cardiac crescent stage (E7.5), such mice show decreased expression of *Nkx2-5*; and a day before that—at primitive streak stage (E6.5)—reduced or delayed expression of the earliest-known marker of cardiogenic mesoderm, *Mesp1*. These data suggest that the ASDs that arise in *Nipbl*-deficient embryos may have their origin in events as early as gastrulation. This is in good agreement with findings in *nipbl*-deficient zebrafish, in which the origin of several heart defects could be traced to the initial migration of cardiogenic mesoderm [23]. It also supports studies that suggest that CHDs frequently have early developmental origins [51,68,71–73].

Early heart development involves interactions among all three germ layers of the embryo: the mesoderm, which produces cardiomyocytes, endocardium, epicardium, and vascular endothelium; the endoderm, which forms an essential substrate along which cardiomyocyte progenitors migrate and proliferate as they form the cardiac crescent; and the ectoderm, which is the source of neural crest cells that contribute to the cardiac cushion and outflow tract [64]. To sort out the relative contributions of different lineages to the production of ASDs in *Nipbl*-deficient mice, we took advantage of recent improvements in gene-trap technology (Fig 2) to selectively create, or rescue, *Nipbl* haploinsufficiency in cardiomyocytes, endoderm and endocardium/endothelium, neural crest, or non-selectively throughout the entire embryo. The results (Figs 5 and 6) implicated both the *cTnt* (cardiomyocyte) and *Sox17* (endoderm and endocardial/endothelial lineages), but not the neural crest. An absence of a role for neural crest is consistent with its fairly late contribution to the heart (Fig 4F), relative to the early molecular abnormalities that occur in the hearts of *Nipbl*-deficient mice (e.g., Fig 1F and 1G).

Surprisingly, we found that creating *Nipbl* haploinsufficiency in either the *cTnt* lineage, the *Sox17* lineage, or the entire embryo produced ASDs at the same frequency (Fig 5). Even more surprisingly, we found that rescue of *Nipbl* haploinsufficiency in either of these two lineages in an embryo that is otherwise globally *Nipbl*-deficient rescued those ASDs (Fig 6). These results—in one case either lineage being sufficient to produce defects and in the other case neither lineage being sufficient to do so—imply that an additional determinant of CHD risk lies outside of both lineages (and thus, most likely, outside both the heart and endoderm). That determinant would have to be protective when the lineage responsible for it is *Nipbl*-deficient (as all non-Cre-expressing lineages are *Nipbl*-deficient in conditional rescue experiments), but it must be only partially protective in order to explain why globally *Nipbl*-deficient embryos do get ASDs.

We speculate that this determinant may be heart size: *Nipbl*-deficient embryos are significantly smaller than wildtype littermates, and so are their hearts. Moreover, heart size is clearly determined by the *Nipbl* genotype of the rest of the body, not the genotype of the cells of the *cTnt* and *Sox17* lineages (Fig 7). The idea that having an abnormally small heart might lower the risk of CHD may seem counterintuitive, but it makes sense if we think of heart development as a process in which a limited pool of progenitor cells must generate a large number of differentiated cells in a short period of time. Under such conditions, a shortfall in cell production might be easier to tolerate if the heart that needs to be built is a smaller one.

This view fits with much of what we know about heart development. The heart is the earliest organ system to become truly functional, and even short delays in cell production tend to be lethal (for example, a 50% deficiency in developing cardiomyocytes at E9.5, due to cardiac-specific deletion of the growth-promoting gene *Yap*, leads to embryo death within one day [74]). In mammals and birds, the need to rapidly remodel the initially simple linear heart tube into an elaborate, four-chambered structure requires the addition of large numbers of cells, many of which arrive by migration from the second heart field (SHF), a collection of cardiac progenitor cells that lie outside the heart tube, and which have been adapted to undergo prolonged proliferation [32,75,76]. Recent studies show the SHF provides most of the cells that drive the early expansion of the heart, including cells that add to ventricles and atria and form both the atrial septum and parts of the ventricular septum [32,34,76].

Interestingly, the SHF gives rise to almost the entire right ventricle [76], the same ventricle that is disproportionately small in *Nipbl*^+/-^ hearts (Fig 1). This result is consistent with a model in which the output of the SHF is reduced in *Nipbl*–deficient embryos. An insufficiency of cardiac progenitors would also be consistent with the reduced expression of *Nkx2-5* and *Mesp1* expression that we observe in early *Nipbl*–deficient embryos. Furthermore, it would fit with results in *nipbl*-deficient zebrafish, in which depletion of cardiac progenitors seems to be the result of an insufficiency of endoderm along which such progenitors migrate [23]. Interestingly, in that system, restoring the number of endoderm cells by overexpression of endoderm-specific transcription factors rescues several heart defects.

One appealing feature of a model in which *Nipbl* deficiency drives progenitor cell numbers to a point at which a large heart cannot always form properly, but a small heart can, is that this model could potentially explain why the incidence of ASDs in conditional *Nipbl*-deficient mice is always ~30%, regardless of whether *Nipbl*-deficiency occurs in the *cTnt* or *Sox17* lineages individually or throughout the entire embryo. It may simply be that, in globally *Nipbl*-deficient animals, effects of reduced *Nipbl* in the *cTnt* lineage and the *Sox17* lineage do interact additively, but we do not observe a stronger phenotype because of the protective effect of small heart size in such mice, which exerts a phenotypic effect in the opposite direction. Future experiments will be needed to verify this conjecture.

Overall, a consistent explanation for all of the conditional mutant phenotypes in the present study is that *Nipbl* haploinsufficiency, in either the cardiac mesoderm or the endoderm upon which it migrates (or both), leads to defective expansion of cardiac progenitors, with an ultimate impact on cardiac morphogenesis that depends on the demands imposed by the rest of the embryo on final heart size. Validating such an explanation will ultimately require measurements of cell numbers and proliferation rates in very early embryos that are beyond the scope of the present study. In addition, the present study did not evaluate possible contributions of epicardium (which derives from neither the *cTnt* or *Sox17* lineages) to *Nipbl* mutant phenotypes, although the timing at which epicardium appears during development suggests it could not explain the early gene expression abnormalities described here (e.g., those in Fig 1F and 1G). Furthermore, since this study focused primarily on ASD, we cannot comment on whether similar multilineage interactions are likely to play a role in other kinds of cardiac defects.

Nevertheless, the present work calls attention to the fact that the embryonic lineages responsible for organogenesis can interact in ways both direct and indirect—global enforcement of scaling relationships being an example of the latter. A consequence of indirect interaction is that determinants of risk for developmental defects can easily turn up in unexpected places, as they did here. This point may need to be kept in mind as efforts continue to be made to discover the genetic causes of human CHDs.

## Materials And Methods

### Animals

#### Ethics statement

All animals were handled in accordance with approved procedures as defined by the National Institutes of Health, and all animal work was approved by the Institutional Animal Care and Use Committee of the University of California, Irvine. For collection of mouse tissues, pregnant dams were humanely killed by CO_2_ anesthesia followed by cervical dislocation. Noon on the day of vaginal plug appearance was designated embryonic day 0.5 (E 0.5). For young embryos (E7–E13.5), embryos were dissected in ice-cold PBS and tissue collected for processing. For E17.5 mouse fetuses, fetuses were removed from the uterus, placed on ice and immediately decapitated, then heart and other tissues removed for further study.

#### Genotyping

All mice and mouse embryos were genotyped by polymerase chain reaction (PCR) using genomic DNA obtained from tail biopsies or tissues taken post-dissection. Primer sequences and PCR conditions for all genotyping performed in these studies is detailed in S2 Table.

#### Mouse lines

*Nipbl*^*+/-*^*line*: The *Nipbl* ^*+/-*^ mouse line (*Nipbl*^*Gt(RRS564)Byg*^, MGI:4332250: *Nipbl*^*+/-*^) was maintained on an outbred CD-1 (Charles River) background. Male *Nipbl* ^*+/-*^ mice were naturally bred with females to obtain embryos used in these studies.

*Nipbl*^*FLEX/+*^*line*: The *Nipbl*^*Gt(EUCE313f02)Hmgu*^ (MGI:4374347) embryonic stem (ES) cell line was purchased from the European Conditional Mouse Mutagenesis Program (EUCOMM). The FLEX genomic modification [33,53,54] to trap and inactivate the *Nipbl* gene, and successful Cre-mediated recombination of the gene-trap allele to functional conformation, were confirmed in vitro by transiently transfecting pTurbo-Cre into *Nipbl*^Gt(EUCE313f02)Hmgu^ ES cells ([77,78]; S2 Fig). Verified ES cells were injected into C57Bl6/J (Jackson Laboratories) blastocysts to generate chimeric male mice. Chimeras were mated with CD-1 females to obtain *Nipbl*^*FLEX/+*^ progeny, which are phenotypically mutant (Fig 2B). *Nipbl*^*FLEX/+*^ mice were maintained on the CD-1 background.

*Nipbl*^*Flox/+*^*line*: *Nipbl*^*FLEX/+*^ male mice were bred with *Actin-FlpE* females (Tg(ACTFLPe)9205Dym, MGI:2448985, Jackson Laboratories)[79] to obtain progeny in which the *Nipbl*^*FLEX*^ allele was recombined to generate mice carrying the *Nipbl*^*Flox*^ allele, in which LoxP and lox5171 recognition sites remained available for Cre-mediated recombination. Resulting *Actin-FlpE/+;Nipbl*^*Flox/+*^ progeny were crossed with C57Bl6/J mice to segregate the recombined allele (*Nipbl*^*Flox*^), and *Nipbl*^*Flox/+*^ mice were maintained thereafter on the C57Bl6/J background. *Nipbl*^*Flox/+*^ and *Nipbl*^*Flox/Flox*^ mice—now >10 generations on C57Bl6/J—are phenotypically wildtype (Fig 2C).

*Nipbl*^*Flrt/+*^*line*: *Nipbl*^*FLEX/+*^ male mice were bred with *Nanog-Cre* mice to generate *Nipbl*^*Flrt/+*^ mice, in which the original *Nipbl*^*FLEX*^ allele was recombined and Flp recognition sites (Frt and F3) remained in active conformation. To isolate the *Nipbl*^*Flrt*^ allele from the *Nanog-Cre* allele, double heterozygous mice were crossed with CD-1 mice. *Nipbl*^*Flrt/+*^ mice are phenotypically wildtype (Fig 2D). This line has not been maintained.

*Nipbl*^*FIN*^*line*: *Nipbl*^*Flox/+*^ mice were bred with *Nanog-Cre* to generate *Nipbl*^*FIN/+*^ mice. *Nipbl*^*FIN/+*^ mice are *Nipbl*-deficient and phenotypically similar to *Nipbl*^*+/*^ and *Nipbl*^*FLEX/+*^mice (Fig 2E). This line has not been maintained.

*Recombinase and reporter mouse lines*: The *cTnt-Cre* line (Tg(Tnnt2-cre)5Blh, MGI:2679081, a gift from Dr. K. Jiao, (Univ. of Alabama, Birmingham, [56]) was maintained on the CD-1 background. *Nanog-Cre* mice [60] were a gift of Dr. A. Economides, Regeneron Pharmaceuticals, and were maintained on C57BL/6J background. *Sox17-2A-iCre* mice (*Sox17*^*tm2*.*1(icre)Heli*^, MGI:4418897 [57]) and *FoxA2-2A-iCre* mice (*Foxa2*^*tm1*.*1(icre)Hri*^, MGI: 5426440; [58]) were generated by Dr. Heiko Lickert (Helmholtz Institute, Munich) and were provided to us by Dr. Mario Cappecchi’s laboratory (University of Utah). Both lines were maintained on C57BL/6J. *Wnt1-Cre* mice (Tg(Wnt1-cre)11Rth, MGI:2386570) were a gift from Dr. David Rowitch (U.C. San Francisco [59]), and were maintained on a CD-1 background. The *Nkx2-5-Cre* line (*Nkx2-5*^*tm1(cre)Rjs*^, MGI:2654594, [55]) was a gift from Dr. R. Schwartz (Baylor College of Medicine). *Nkx2-5-Cre* mice were obtained on a mixed background and maintained on C57BL/6J. The *Actin-FlpE* line (Tg(ACTFLPe)9205Dym, MGI:2448985 [79]) was obtained from the Jackson Laboratory and maintained on C57BL/6J. The reporter line used in these studies was *Td-tomato-EGFP* (*Gt(ROSA)26Sor* ^*tm4(ACTB-tdTomato*,*-EGFP)Luo*^, MGI:3716464 [61]). Homozygous *Td-tomato-EGFP* mice on C57BL/6J were purchased from the Jackson Laboratory and maintained by interbreeding.

#### Generation of mouse embryos for testing tissue-specific depletion and rescue of *Nipbl* expression

To generate embryos for *Nipbl* depletion experiments, *Nipbl*^*Flox/+*^ mice were naturally mated with mice carrying any one of several Cre transgenes (*Nanog-Cre*, *Sox17-2A-iCre*, *FoxA2-2A-iCre*, *Wnt1-Cre*, or *cTnt-Cre*). To generate embryos for *Nipbl* rescue experiments, cryopreserved *Nipbl*^*FLEX/+*^ sperm was used for in vitro fertilization (IVF) of eggs from superovulated females of indicated mouse lines heterozygous for *Cre-*expressing transgenes. Sperm cryopreservation and IVF procedures were carried out by the UCI Transgenic Mouse Facility using published procedures [80]. Because Cre-driver mice were not available on uniform genetic backgrounds, potential genetic background effects in these experiments were minimized by making comparisons within litters, or within groups of litters in which the distribution of genotypes across the litters was as uniform as possible.

### Histochemistry, In Situ Hybridization, and Microscopy

Whole mount X-gal staining on E7.75–13.5 mouse embryos for detecting β-galactosidase activity was performed as described [81]. Histological evaluations were performed on tissue fixed in 4% paraformaldehyde (PFA) in phosphate buffer or neutral buffered formalin (VWR 16004–126). Hematoxylin and Eosin Y (H&E) staining was performed on 20 μm cryosections (for E13.5 hearts) or 7 μm paraffin sections (for E17.5 hearts) using standard techniques. E17.5 hearts were positioned at a canonical angle for analysis in histological sections and MRI studies: atria superior to ventricles, with the apex of the heart inferior and the left atrium and ventricle to the right; sections were assessed from most ventral to most dorsal.

Whole mount ISH on E7.5–10.5 embryos was performed as described [82], except that for E7.5 embryos, incubation time in proteinase K was reduced to 5 min. RNA probes for ISH were generated as follows: 400 bp of *Nkx2*.*5* transcript (997–1,396 bp of ENSMUST00000015723); 353 bp of mouse *Hand1* transcript (669––1,021 bp of ENSMUST00000036917); and 901 bp of mouse *Mesp1* transcript (62–962 bp of ENSMUST00000030544).

Whole mount ISH, X-gal, and fluorescent images, as well as all images of paraffin-sectioned hearts, were obtained using a Discovery V8 stereomicroscope equipped with Axiovision software (Zeiss). Confocal microscopy was performed on 30 μm sections of E15.5 hearts. Images were taken every 5 μm using a 20x 0.75NA Olympus objective on an Olympus Fluoview FV1000 microscope, and processed and stitched using Python and ImageJ Software as described [83].

### Magnetic Resonance Imaging (MRI)

Torsos from E17.5 embryos containing intact cardiopulmonary organs (heart and lungs) were fixed in neutral buffered formalin for a minimum of 1 wk at 4°C. Intact torsos, or torsos with ribs removed, were rinsed with phosphate buffered saline (PBS; 2 x 5 min.) and soaked in 2.5 mM Gadoteridol (Gd-HP-DO3A, a.k.a. ProHance, Braccoo Diagnostics Inc., Princeton, NJ) in PBS for 12 h at 4°C. After soaking, samples were embedded in 2% low melting point (LMP) agarose (Sea Plaque GTG Agarose, FMC Bioproducts) and stacked in a 20 mm diameter glass tube. The chamber was then filled with perfluoro-polyether Galden-D (Inland Vacuum Industries, Churchville, New York) to limit tissue dehydration as well as susceptibility effects at the surface of the specimen. Imaging was performed in a vertical bore 11.7T (500 MHz) Bruker AVANCE imaging spectrometer with a microimaging gradient insert and 20 mm birdcage RF coil. Images were acquired with a 3D RARE protocol (TR/TE_effective_, 250 ms/16 ms) with a RARE factor of 4 [84], voxel size of 50^3^ μm^3^ with typical image matrix: 512x320x320 and field of view (FOV): 25.6x16x16 mm^3^, and number of acquisitions = 10–18. The FOV and matrix sizes were modified to accommodate differing numbers of samples and sample sizes. Scanning was performed at 15°C to minimize noise.

Each heart from a scan was isolated as a file of 80^3^ pixel^3^ using ImageJ (v. 1.45s). Heart scans were evaluated for the presence or absence of ASD, VSD and/or PTA using the volume viewer plug-in of ImageJ. Hearts were scored positive for ASD when: 1) a minimum of two consecutive sections (100 microns) lacked atrial septum; 2) gap in atrial septum tissue was greater than 3 pixels (150 microns); and 3) growth of both atrial septum walls (septum primum and septum secundum) were stunted (observed as flat) at both the superior and inferior ends of the developing atrial septum at the region of the atrio-ventricular valves. Hearts were scored positive for VSD when ventricular septum was not continuous in one or more sections of the MRI scan. Hearts were scored positive for PTA when the aorta and pulmonary artery were observed as fused. Ventricular volumes were calculated from MRI data using the outline function of ImageJ: The outlined ventricle area was calculated for each section and multiplied by section thickness (section thickness = 1 pixel or 50 μm); total ventricular volume for each heart was calculated as the sum of these volumes. Heart defects detected by MRI were confirmed by paraffin sectioning and histological staining. Chi-square test was used to calculate *p*-values for the frequency of heart defects (ASD, VSD, and/or PTA) at E17.5 for all crosses. Mann-Whitney U test was used to calculate *p*-values for measurements of the great vessels, ventricle wall thickness, and ventricular volume at E17.5.

### Optical Projection Tomography

E10.5 embryos were fixed in 4% PFA, washed thoroughly in PBS and embedded in LMP agarose. Agarose blocks were affixed to metal mounts with cyanoacrylate Krazy Glue, trimmed, dehydrated through three changes of methanol for 8–12 h, and then immersed in 2:1 benzyl benzoate:benzyl alcohol mixture until optically clear (minimum 6 h). Specimens were scanned using an optical projection tomography scanner (OPT 3001M; Bioptonics, United Kingdom), under ultraviolet light using a GFP1 filter (exciter 425 nm/40 nm, emitter LP475 nm). Tomographic reconstruction was carried out using NRecon v. 1.6.1 (Skyscan, Belgium) to generate a series of bitmap images. Three-dimensional reconstructions were rendered from stacked bitmaps using ImageJ v. 1.41o (http://resb.info.nih.gov/ij) and Amira v. 5.2.2 (Visage Imaging, USA).

### Quantitative RT-PCR

RNA extraction was performed using an Aurum Total RNA kit (BioRad, USA) according to the manufacturer’s instructions. RNA was reverse-transcribed into cDNA using oligo dT and random hexamers with Superscript II reverse transcriptase (Invitrogen). Quantitative real-time PCR amplifications were performed in an iQ5 iCycler real time PCR detection system (Bio-Rad) using the iQ SYBR Green supermix (Bio-Rad). qRT-PCR was performed with primer sets given in S1 Table, using the following cycles: an initial cycle at 95°C for 3 min, followed by 40 cycles of 95°C for 10 s each, 59°C for 30 s, and 72°C for 30 s, followed by melt- curve analysis from 61–95°C in 0.5°C increments. mRNA expression was normalized to expression of *B-2-microgoblulin* (*B2m*) or *glyceraldehyde 3-phosphate dehydrogenase* (*GAPDH*), and the relative expression of target genes was obtained by the 2^-ddCt^ method [85]. A range of 3–14 biological replicates (individual hearts between the ages of E10-13.5, brain or kidney at E17.5) were assessed in technical duplicates or triplicates for each gene. Statistical significance was determined using Student’s *t* test (with Bonferroni correction where indicated).

## Supporting Information

S1 Data Sample numbers and all quantitative observations underlying the data summarized in all Figures and Supporting Information Figures. (XLSX)

S1 Table Primer Sequences. The following primer sequences were used in Q-RT-PCR experiments to detect mRNA expression levels of 29 cardiac developmental genes, plus Nipbl and beta-2 microglobulin.
(XLSX)

S2 Table Summary of transgenic mouse lines and PCR primers and conditions used for genotyping. (DOCX)

S1 Fig Heart development genes showing no significant change in expression in Nipbl+/- heart versus wildtype controls. Q-RT-PCR was performed as described in Materials and Methods, and S1 Table. Values for each tested gene were normalized to expression value of *B-2-microgoblulin* (*B2M*) in the same PCR run, and relative expression of tested genes was obtained by the 2^-ddCt^ method as described in Materials and Methods. 3–14 biological replicates (individual hearts) were assayed in duplicate for each gene (see S1 Data). Data are expressed as mean (± SEM); statistical significance was determined using Student’s *t-*test with Bonferroni correction. No gene tested showed a significant difference in *Nipbl*^*+/-*^ hearts versus wildtype controls, except *Nipbl*, which was tested at E10.5 (52%, P<0.001).
(TIF)

S2 Fig Conditional-invertible gene modifications of the *Nipbl* FLEX allelic series. A. Schematic of EUCE313f02 (*Nipbl*^*FLEX*^) allele from which the *Nipbl* ^*FLEX/+*^ mouse line and subsequent *Nipbl*^*Flox/+*^, *Nipbl*^*Flrt/+*^, and *Nipbl*^*FIN/+*^ mouse lines are derived using Cre and Flp DNA-recombinases). Top: The SA-βgeo-pA (rsFlp-Rosa-bgeo) cassette in the *Nipbl*^*FLEX*^ allele traps *Nipbl* expression after Exon1, and instead expresses the *β-geo* reporter gene resulting in a null allele. Middle: Flp recombinase (right path) inverts the SA-βgeo-pA cassette at frt and F3 sites, simultaneously excising the heterotypic recognition targets, and locking the cassette against reinversion, resulting in the *Nipbl*^*Flox*^ allele. The same principle is adaptable to Cre recombinase, using the LoxP and lox5171 sites, resulting in the *Nipbl*^*Flrt*^ allele (left path). Either (Flp or Cre mediated) inversion activates normal splicing between the endogenous splice sites (Exon 1 to Exon 2), skipping the inverted SA-βgeo-pA cassette, thereby repairing the mutation and resulting in a phenotypically-wildtype allele. Bottom: Subsequent Cre (right path) or Flp (left path) recombinase-mediated inversion repositions the SA-βgeo-pA cassette, activating the gene-trap again, and re-introducing the mutation, resulting in the phenotypically null *Nipbl*^*FIN*^ allele. Each allele can be distinguished by genomic PCR, in which primers are designed within the rsFlp-Rosa-βgeo cassette (across the *β-geo* reporter and the arm of cassette outside of the recognition sites; white: B050, gray: B048, and black: B045 arrows). Each genomic conformation yields a PCR amplicon of a different size (*Nipbl*^*FLEX*^: 652 bp, *Nipbl*^*Flox*^: 782bp, *Nipbl*^*Flrt*^: 735 bp, and *Nipbl*^*FIN*^: 518 bp, also see S2 Table). Frt (purple triangles) and F3 (green triangles) are heterotypic target sequences for FLP recombinase; loxP (orange triangles) and lox5171 (yellow triangles) are heterotypic target sequences for Cre-recombinase; SA, splice acceptor; β-geo, β-galactosidase/neomycin phosphotransferase fusion gene; pA, bovine growth hormone polyadenylation sequence. B. Schematic showing the location of PCR primers to detect the wildtype *Nipbl* allele (black and blue arrowheads show *Nipbl* forward *Nipbl* reverse primers, respectively; 492 bp amplicon) and any of the *Nipbl*^*Gt(EUCE313f02)Hmgu*^ derivatives (*Nipbl*^*Gt*^) containing the rsFlp-Rosa-βgeo cassette (red arrowhead shows 313f02 forward primer, blue arrowhead, *Nipbl* reverse primer; 302 bp amplicon). C. To ensure that the rsFlp-Rosa-βgeo cassette can undergo recombinase-mediated inversion, EUC313f02 *Nipbl*^*FLEX/+*^ ES cells were electroporated with pTurbo-Cre to convert the *Nipbl*^*FLEX*^ allele to the *Nipbl*^*Flrt*^ conformation in vitro. After the pTurbo-Cre plasmid was electroporated into ES cells, clonal colonies were isolated and screened by PCR genotyping, X-gal staining, and sensitivity to G418 to identify *Nipbl*^*Flrt/+*^ clones. For X-gal staining, ES cells were fixed for 10 minutes in 2 mM MgCl_2_, 0.5% glutaraldehyde in 1X PBS, followed by a 30 minute wash (2 mM MgCl_2_ in 1xPBS), and permeabilization (0.2 mM MgCl_2_, 0.1% Triton X-100, 0.01% deoxycholate in 1xPBS), all at room temperature. X-gal staining (5 mM K3Fe(CN)_6_, 5 mM K4Fe(CN)_6_, 2 mM MgCl_2_, 0.1% Triton X-100, 0.01% deoxycholate, 1 mg/mL X-gal) was performed at 37°C until blue precipitate was detected. For G418 selection, ES cells were treated with 0.1mg/ml of G418 (Gibco) in E14TG2A medium (protocol available at https://www.eummcr.org/protocols/tissue-culture_e14tg2a.pdf) containing 1000 U/ml leukemia inhibitory factor (LIF: ESGRO Millipore, Massachusetts, USA). Medium was changed daily for 8 days. Only *Nipbl*^*FLEX/+*^ ES cells were positive for X-gal staining and resistant to G418 treatment. In contrast, E14Tg2A (wildtype) and *Nipbl* ^*Flrt/+*^ ES cells were not stained by X-gal and cells were killed by G418 exposure. Scale bar = 500 μm. D. Q-RT-PCR results showing reduced *Nipbl* expression in *Nipbl*^*FLEX/+*^ ES cell clones, and normal *Nipbl* expression levels in *Nipbl*^*FLEX/+*^ ES cell clones transfected with p-Turbo Cre (i.e. *Nipbl*^*Flrt/+*^ clones). *Nipbl* mRNA expression was calculated as the ratio of the Ct value for *Nipbl* normalized to the Ct value for *Gapdh* in the same sample (dCt). Data are shown as 2^-dCt^ (x 10^−2^) for individual ES cell clones; error bars in graph represent the range of 2^-dCt^ values of technical duplicates from each individual ES cell clone. *Nipbl* Q-RT-PCR primers and conditions are described in Materials and Methods, S1 Table and S1 Fig. E. Representative gel showing PCR genotyping for *Nipbl*^*Gt*^ and wildtype alleles. PCR results show *Nipbl* alleles present in *Nipbl*^*Flox/+*^ tissue (WT: 492 bp and Nipbl^Gt^: 302 bp) and wildtype littermates (WT: 492 bp only), using primers shown in B and detailed in S2 Table. F. Representative gel showing PCR results for tissues from mice representing the complete *Nipbl*^*FLEX*^ allelic series (*Nipbl*^*FLEX/+*^: 652 bp, *Nipbl*^*Flox/+*^: 782 bp, *Nipbl*^*Flrt/+*^: 735 bp, and *Nipbl*^*FIN/+*^: 518 bp) using the 3-primer PCR described in A and S2 Table.
(TIF)

S3 Fig Growth retardation and poor survival of *Nipbl*^*FLEX/+*^ mice is rescued in *Nipbl*^*Flox/+*^ and *Nipbl*^*Flrt/+*^mice. A. Growth curves of *Nipbl*^*FLEX/+*^ mice compared to wildtype littermates from post-natal day (P) 1 to P21, from 17 litters. Data are pooled by genotype (wildtype, *Nipbl*^*FLEX/+*^ or not genotyped due to early demise) and survival status (survived to weaning versus died before weaning), from birth to weaning at P21. For clarity, only upper error bars (standard deviation, SD) are shown for wildtype and *Nipbl*^*FLEX/+*^ daily weight averages. Data show that wildtype mice gain weight steadily for the first 21 days of life (black circles), whereas *Nipbl*^*FLEX/+*^ survivors (red open boxes) and non-survivors (blue filled boxes) grow at impaired rates. The weights of *Nipbl*^*FLEX/+*^ mice overlapped within the first few days of life regardless of whether they survived to weaning or not. Growth of un-genotyped non-survivors (green Xs) was similar to *Nipbl*^*FLEX/+*^ non-survivors (blue filled boxes) within the first 12 days of life, suggesting that pups that failed to survive were likely *Nipbl*^*FLEX/+*^. Some pups that were logged as having been born could not be genotyped because tissue could not be recovered. B. Growth curves of *Nipbl*^*Flox/+*^ mice compared to wildtype littermates from P1 to P21, from 5 litters. Data are pooled by genotype. Data show overlapping growth and similar sizes of wildtype and *Nipbl*^*Flox/+*^ mice. For purposes of clarity, only upper error bars for *Nipbl*^*Flox/+*^ mice (SD, blue) and lower error bars for wildtype (SD, black) are shown. C. Growth curves for *Nipbl*^*Flrt/+*^ mice compared to wildtype littermates from P1 to P21, from 4 litters. Data show overlapping growth and similar weights of wildtype and *Nipbl*^*Flrt/+*^ mice. For purposes of clarity, only upper error bars for *Nipbl*^*Flrt/+*^ mice (SD, green) and lower error bars for wildtype (SD, black) are shown.
(TIF)

S4 Fig *Nipbl*^*FLEX/+*^ hearts do not display pulmonary stenosis, aortic stenosis, or ventricular hypertrophy. To assess stenosis, diameters of the aorta and pulmonary artery were measured from images using Axiovision software (Zeiss). 90% of pulmonary stenoses are isolated at the valvular region, so diameters were measured just distal to the semilunar valves (supravalvular). To confirm that any stenoses observed did not involve the pulmonary trunk/aorta, serial sections were followed by the observer to the bifurcation. To assess hypertrophic cardiomyopathy, ventricular wall thickness was measured and the ratio of the right to left wall thickness was calculated for each sample, and different genotypes were compared. Diameter of the ventricular wall (left and right myocardium, excluding trabeculae) was measured in images of H&E stained paraffin sections in the region that was midway through the atrioventricular valve (ventral-dorsal) and midway through the ventricular septum (superior to inferior). A. The supravalvular diameter of the great arteries of paraffin sectioned wildtype and *Nipbl*^*FLEX/+*^ hearts were measured as indicated by black bar. B. Both the pulmonary artery diameter and the aorta diameter (C) were significantly smaller in *Nipbl*^*FLEX/+*^ hearts (N = 11) compared to wildtype littermates (N = 9) (Mann-Whitney U, P<0.05). D. The thickness of the ventricular walls (excluding trabiculae) were measured as indicated by black bar. Ventricular wall thickness was significantly reduced in *Nipbl*^*FLEX/+*^ hearts (N = 11) compared to wildtypes (N = 9) on both the right (E) and left (F) walls (Mann-Whitney U, P<0.025). G. A ratio of the right to left ventricle wall thickness revealed no significant difference between wildtypes and *Nipbl*^*FLEX/+*^ mice (Mann-Whitney U P>0.05). Differences observed in C, D, E, and F likely due to the overall smaller body and heart size observed in *Nipbl*^*FLEX/+*^ embryos (see results and Fig 7). LV, left ventricle; PA, pulmonary artery; RV, right ventricle.
(TIF)

S5 Fig *Nkx2-5*^*Cre/+*^ hearts have half the normal level of *Nkx2-5* expression. Q-RT-PCR was performed on E10.5 *Nkx2-5*^*+/+*^ (N = 7) and *Nkx2-5*^*Cre/+*^ (N = 5) hearts. *Nkx2-5*^*Cre/+*^ hearts had approximately half the *Nkx2-5* expression of *Nkx2-5*^*+/+*^ littermates. *Nkx2-*5 mRNA expression was normalized to expression of *B2m*, and the relative expression of *Nkx2-5* was obtained by the 2^-ddCt^ method; data are expressed as mean ± SEM. PCR primers and conditions are described in Materials and Methods, and S1 Table. Asterisk: Student’s t-test, P = 0.001.
(TIF)

## Abbreviations

ASD: atrial septal defect
CdLS: Cornelia de Lange Syndrome
CHD: congenital heart defect
ES: embryonic stem
FLEX: Flip-Excision
ISH: in situ hybridization
MRI: magnetic resonance imaging
Nipbl: Nipped-B homologue
OPT: Optical projection tomography
qRT-PCR: Quantitative reverse transcription PCR
VSD: ventricular septal defect
