# Supplementary material for: Conditional Creation and Rescue of Nipbl-Deficiency in Mice Reveals Multiple Determinants of Risk for Congenital Heart Defects
Source: PLoS Biol. 2016 Sep 8;14(9):e2000197. doi: 10.1371/journal.pbio.2000197 (PMC5016002; doi:10.1371/journal.pbio.2000197)
Supplement: S2 Table — (DOCX) [file pbio.2000197.s003.docx]

| **Mouse Line** | **Primers** | **PCR Condition** |
| --- | --- | --- |
| *Nipbl^+/-^*  *Nipbl* ^Gt(RRS564)Byg^, MGI:4332250 | LacZ Forward 5’- TGATGAAAGCTGGCTACAG-3’  LacZ Reverse 5’-ACCACCGCACGATAGAGATT-3’  (see Ref. 1 below)  *β-geo*-500 bp | LacZ PCR  94°C (30 sec); 58°C (30 sec); 72°C (30 sec) 30 cycles |
| *Nipbl FLEX/+*  *Nipbl* ^Gt(EUCE313f02)Hmgu^, MGI:4374347 | LacZ Forward 5’- TGATGAAAGCTGGCTACAG-3’  LacZ Reverse 5’-ACCACCGCACGATAGAGATT-3’  (see Ref. 1 below)  *β-geo*-500 bp | LacZ PCR  94°C (30 sec); 58°C (30 sec); 72°C (30 sec) 30 cycles |
|  | B045 5’- CTCCGCCTCCTCTTCCTC CATC-3’  B048 5’- CCTCCCCCGTGC CTTCCTTGAC-3’  B050 5’- TTTGAGGGGACGACGACAGTCT-3’  (see Ref. 2 below)  FLEX conformation-652 bp | General FlEx gene conformation PCR  94°C (1 min); 59°C (30 sec); 72°C (1min) 30 cycles |
|  | Nipbl Forward 5’-TTCTCAGCTTCCACGTTTCA-3’  313f02 Forward 5’- GAGCCCCCAAATGAAAGAC-3’  Nipbl Reverse 5’- CACCCCTTATGAATAAGTAACAGTTC-3’  *Nipbl* Gt (gene trap; EUCOMM) allele- 302 bp  *Nipbl* wildtype allele- 492 bp | *Nipbl* ^Gt^ specific PCR    94°C (1 min); 59°C (1 min); 72°C (1 min) 30 cycles |
| *Nipbl Flox/+*  *Nipbl* ^Gt(EUCE313f02)1.1Hmgu^ | LacZ Forward 5’- TGATGAAAGCTGGCTACAG-3’  LacZ Reverse 5’-ACCACCGCACGATAGAGATT-3’  (see Ref. 1 below)  *β-geo*-500 bp | LacZ PCR  94°C (30 sec); 58°C (30 sec); 72°C (30 sec) 30 cycles |
|  | B045 5’- CTCCGCCTCCTCTTCCTC CATC-3’  B048 5’- CCTCCCCCGTGC CTTCCTTGAC-3’  B050 5’- TTTGAGGGGACGACGACAGTCT-3’  (see Ref. 2 below) *Nipbl* Flox conformation-782 bp | FlEx gene conformation PCR  94°C (1 min); 59°C (30 sec); 72°C (1min) 30 cycles |
|  | Nipbl Forward 5’-TTCTCAGCTTCCACGTTTCA-3’  313f02 Forward 5’- GAGCCCCCAAATGAAAGAC-3’  Nipbl Reverse 5’- CACCCCTTATGAATAAGTAACAGTTC-3’  *Nipbl* Gt (gene trap; EUCOMM) - 302 bp  *Nipbl* wildtype allele- 492 bp | *Nipbl* ^Gt^ specific PCR    94°C (1 min); 59°C (1 min); 72°C (1 min) 30 cycles |
| *Nipbl Flrt/+* | LacZ Forward 5’- TGATGAAAGCTGGCTACAG-3’  LacZ Reverse 5’-ACCACCGCACGATAGAGATT-3’  (see Ref. 1 below)  *β-geo*-500 bp | LacZ PCR  94°C (30 sec); 58°C (30 sec); 72°C (30 sec) 30 cycles |
|  | B045 5’- CTCCGCCTCCTCTTCCTC CATC-3’  B048 5’- CCTCCCCCGTGC CTTCCTTGAC-3’  B050 5’- TTTGAGGGGACGACGACAGTCT-3’  (see Ref. 2 below) *Nipbl Flrt* conformation-735 bp | FlEx gene conformation PCR  94°C (1 min); 59°C (30 sec); 72°C (1min) 30 cycles |
|  | Nipbl Forward 5’-TTCTCAGCTTCCACGTTTCA-3’  313f02 Forward 5’- GAGCCCCCAAATGAAAGAC-3’  Nipbl Reverse 5’- CACCCCTTATGAATAAGTAACAGTTC-3’  *Nipbl* Gt (gene trap; EUCOMM) - 302 bp  *Nipbl* wildtype allele- 492 bp | *Nipbl* ^Gt^ specific PCR    94°C (1 min); 59°C (1 min); 72°C (1 min) 30 cycles |
| *Nipbl FIN/+* | LacZ Forward 5’- TGATGAAAGCTGGCTACAG-3’  LacZ Reverse 5’-ACCACCGCACGATAGAGATT-3’  (see Ref. 1 below)  β-geo-500 bp | LacZ PCR  94°C (30 sec); 58°C (30 sec); 72°C (30 sec) 30 cycles |
|  | B045 5’- CTCCGCCTCCTCTTCCTC CATC-3’  B048 5’- CCTCCCCCGTGC CTTCCTTGAC-3’  B050 5’- TTTGAGGGGACGACGACAGTCT-3’  (see Ref. 2 below) *Nipbl FIN* conformation-518 bp | FlEx gene conformation PCR  94°C (1 min); 59°C (30 sec); 72°C (1min) 30 cycles |
|  | Nipbl Forward 5’-TTCTCAGCTTCCACGTTTCA-3’  313f02 Forward 5’- GAGCCCCCAAATGAAAGAC-3’  Nipbl Reverse 5’- CACCCCTTATGAATAAGTAACAGTTC-3’  *Nipbl* Gt (gene trap; EUCOMM) - 302 bp  *Nipbl* wildtype allele- 492 bp | *Nipbl* ^Gt^ specific PCR    94°C (1 min); 59°C (1 min); 72°C (1 min) 30 cycles |
| *cTnt*-Cre  Tg(*Tnnt2*-cre)5Blh, MGI:2679081 | C1 Forward 5’- GCACTGATTTCGACCAGGTT-3’  C2 Reverse 5’- GCTAACCAGCGTTTTCGTTC-3’  (see Ref. 3 below)  Cre-200 bp | Cre targeted PCR  94°C (30 sec); 62°C (30 sec); 72°C (30 sec) 30 cycles |
| *Foxa2*-2A-iCre  *Foxa2* ^tm1.1(icre)Hri^, MGI: 5426440 | 5-iCre forward 476 5' -CTCTGACAGATGCCAGGACA-3'  3-iCre reverse 478: 5'-TGATTTCAGGGATGGACACA-3'  iCre-459 bp | iCre targeted PCR  94°C (30 sec); 58°C (30 sec); 72°C (30 sec) 30 cycles |
| *Nanog*-Cre | C1 Forward 5’- GCACTGATTTCGACCAGGTT-3’  C2 Reverse 5’- GCTAACCAGCGTTTTCGTTC -3’  (see Ref. 3 below)  Cre-200 pb | Cre targeted PCR  94°C (30 sec); 62°C (30 sec); 72°C (30 sec) 30 cycles |
| *Nkx2-5* Cre/+  *Nkx2-5*^tm1(cre)Rjs^ , MGI:2654594 | C1 Forward 5’- GCACTGATTTCGACCAGGTT-3’  C2 Reverse 5’- GCTAACCAGCGTTTTCGTTC -3’  (see Ref. 3 below)  Cre-200 pb | Cre targeted PCR  94°C (30 sec); 62°C (30 sec); 72°C (30 sec) 30 cycles |
|  | Nkx2-5 Forward 5’- GATTAGCTTAAGCGGAGCTGGGTGTCC -3’  Nkx2-5 Reverse 5’-GTTCTGGAACCAGATCTTGACCTGCTGGGA -3’  Nkx2-5 CRE Rev 5’- GCCGCATAACCAGTGAAACAGCATTGC -3’  (see Ref. 4 below)  *Nkx2-5* Cre-481bp  Wildtype-358 bp | Nkx2.5 allele specific PCR  94°C (30 sec); 59°C (30 sec); 72°C (30 sec) 31 cycles |
| *Sox17-*2A-iCre  *Sox17* ^tm2.1(icre)Heli^, MGI:4418897 | 5-iCre forward 476 5' -CTCTGACAGATGCCAGGACA-3'  3-iCre reverse 478: 5'-TGATTTCAGGGATGGACACA-3'  iCre-459bp | iCre targeted PCR  94°C (30 sec); 58°C (30 sec); 72°C (30 sec) 30 cycles |
| *Wnt1*-Cre  Tg(*Wnt1*-cre)11Rth, MGI:2386570 | C1 Forward 5’- GCACTGATTTCGACCAGGTT-3’  C2 Reverse 5’- GCTAACCAGCGTTTTCGTTC-3’  (see Ref. 3 below)  Cre-200 pb | Cre targeted primers:  94°C (30 sec); 62°C (30 sec); 72°C (30 sec) 30 cycles |
| ActinFlpE  Tg(ACTFLPe)9205Dym, MGI:2448985 | ActFlpe For: 5’-CACTGATATTGTAAGTAGTTTGC-3’  ActFlpe Rev: 5’-CTAGTGCGAAGTAGTGATCAGG-3’  ActFlpe transgene- 725 bp | ActFlpE PCR  94°C (30 sec); 58°C (1 min); 72°C (1 min) 35 cycles |
| td-Tomato-GFP  *Gt(ROSA)26Sor* ^tm4(ACTB-tdTomato,-EGFP)Luo^_,_ MGI:3716464 | oIMR7318 5’- CTCTGCTGCCTCCTGGCTTCT-3’  oIMR7319 5’- CGAGGCGGATCACAAGCAATA-3’  oIMR7320 5’- TCAATGGGCGGGGGTCGTT-3’  (see Ref. 5 below)  *Rosa26* tdTomatoGFP- 250bp  Wildtype-330bp | Targeted primers:  94°C (30 sec); 61°C (60 sec); 72°C (60 sec) 35 cycles |

References:

1. Kawauchi S, Calof AL, Santos R, Lopez-Burks ME, Young CM, Hoang MP, et al. Multiple organ system defects and transcriptional dysregulation in the *Nipbl(+/-)* mouse, a model of Cornelia de Lange Syndrome. PLoS Genet. 2009 PMID: 19763162; PubMed Central PMCID: PMC2730539.
2. Schnütgen F, De-Zolt S, Van Sloun P, Hollatz M, Floss T, Hansen J, et al. Genomewide production of multipurpose alleles for the functional analysis of the mouse genome. Proc Natl Acad Sci U S A. 2005 PubMed PMID: 15870191; PubMed Central PMCID: PMC1129123.
3. Kawauchi S, Kim J, Santos R, Wu HH, Lander AD, Calof AL. Foxg1 promotes olfactory neurogenesis by antagonizing Gdf11. Development. 2009 PubMed PMID: 19297409; PubMed Central PMCID: PMC2674256.
4. Lombardi R, Dong J, Rodriguez G, Bell A, Leung TK, Schwartz RJ, et al. Genetic fate mapping identifies second heart field progenitor cells as a source of adipocytes in arrhythmogenic right ventricular cardiomyopathy. Circ Res. 2009 PubMed PMID: 19359597; PubMed Central PMCID: PMC2767296.
5. https://www2.jax.org/protocolsdb/f?p=116:5:0::NO:5:P5_MASTER_PROTOCOL_ID,P5_JRS_CODE:1143,007576
