# Supplementary figures and images for: Conditional Creation and Rescue of Nipbl-Deficiency in Mice Reveals Multiple Determinants of Risk for Congenital Heart Defects
Source: PLoS Biol. 2016 Sep 8;14(9):e2000197. doi: 10.1371/journal.pbio.2000197 (PMC5016002; doi:10.1371/journal.pbio.2000197)

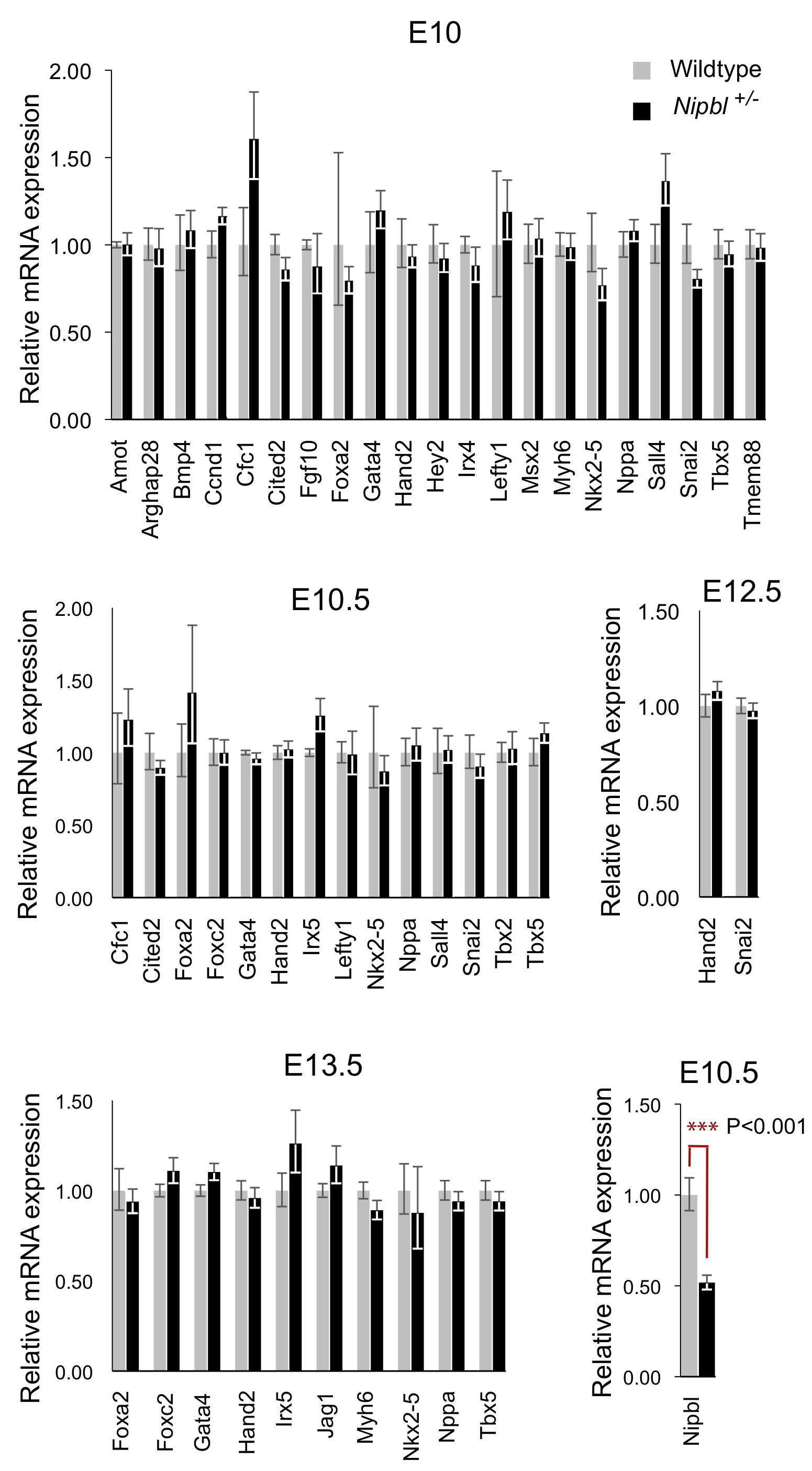

Supplement: S1 Fig — Q-RT-PCR was performed as described in Materials and Methods, and S1 Table. Values for each tested gene were normalized to expression value of B-2-microgoblulin (B2M) in the same PCR run, and relative expression of tested genes was obtained by the 2-ddCt method as described in Materials and Methods. 3–14 biological replicates (individual hearts) were assayed in duplicate for each gene (see S1 Data). Data are expressed as mean (± SEM); statistical significance was determined using Student’s t-test with Bonferroni correction. No gene tested showed a significant difference in Nipbl+/- hearts versus wildtype controls, except Nipbl, which was tested at E10.5 (52%, P<0.001). (TIF) [file pbio.2000197.s004.tif]

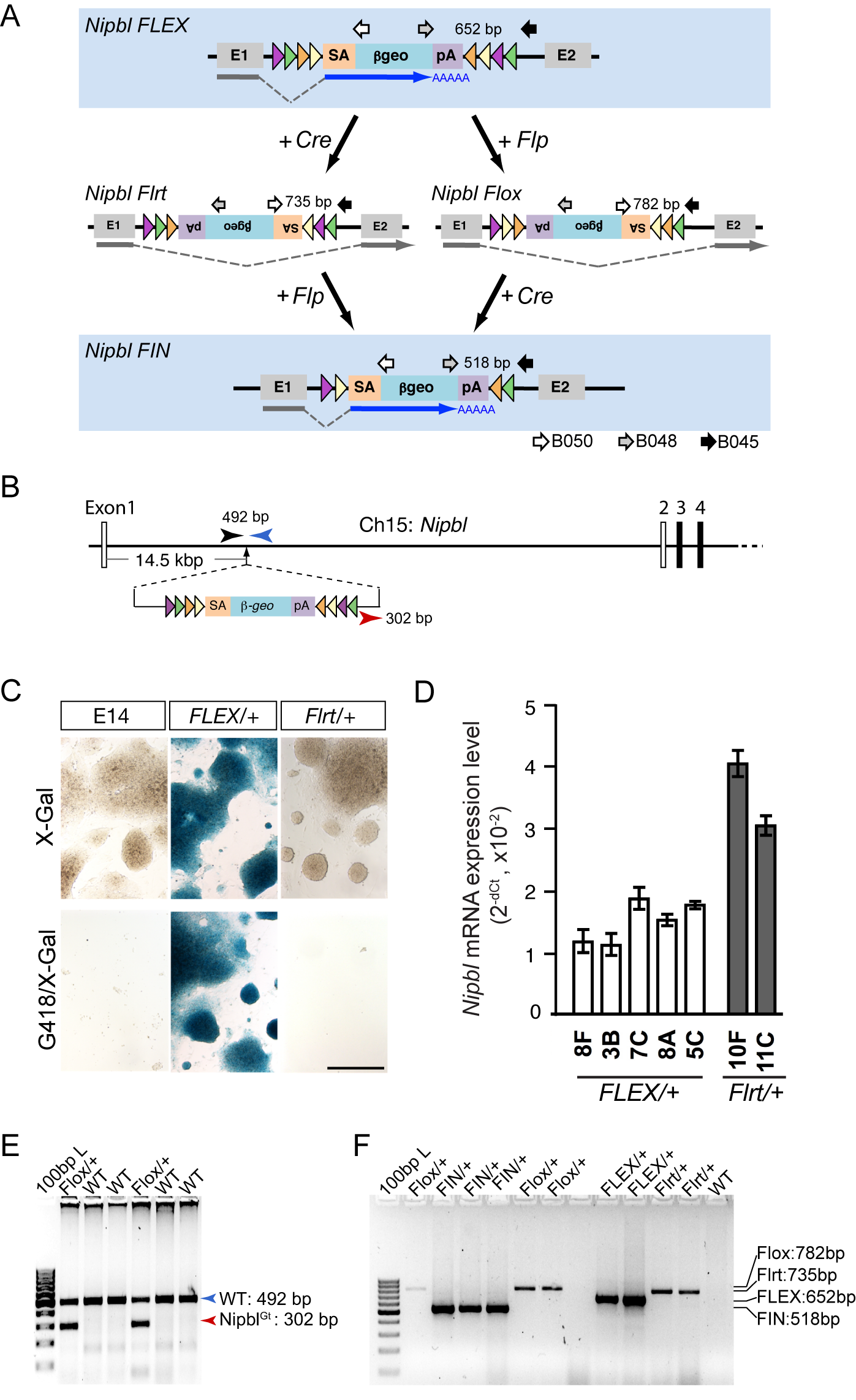

Supplement: S2 Fig — A. Schematic of EUCE313f02 (NipblFLEX) allele from which the Nipbl FLEX/+ mouse line and subsequent NipblFlox/+, NipblFlrt/+, and NipblFIN/+ mouse lines are derived using Cre and Flp DNA-recombinases). Top: The SA-βgeo-pA (rsFlp-Rosa-bgeo) cassette in the NipblFLEX allele traps Nipbl expression after Exon1, and instead expresses the β-geo reporter gene resulting in a null allele. Middle: Flp recombinase (right path) inverts the SA-βgeo-pA cassette at frt and F3 sites, simultaneously excising the heterotypic recognition targets, and locking the cassette against reinversion, resulting in the NipblFlox allele. The same principle is adaptable to Cre recombinase, using the LoxP and lox5171 sites, resulting in the NipblFlrt allele (left path). Either (Flp or Cre mediated) inversion activates normal splicing between the endogenous splice sites (Exon 1 to Exon 2), skipping the inverted SA-βgeo-pA cassette, thereby repairing the mutation and resulting in a phenotypically-wildtype allele. Bottom: Subsequent Cre (right path) or Flp (left path) recombinase-mediated inversion repositions the SA-βgeo-pA cassette, activating the gene-trap again, and re-introducing the mutation, resulting in the phenotypically null NipblFIN allele. Each allele can be distinguished by genomic PCR, in which primers are designed within the rsFlp-Rosa-βgeo cassette (across the β-geo reporter and the arm of cassette outside of the recognition sites; white: B050, gray: B048, and black: B045 arrows). Each genomic conformation yields a PCR amplicon of a different size (NipblFLEX: 652 bp, NipblFlox: 782bp, NipblFlrt: 735 bp, and NipblFIN: 518 bp, also see S2 Table). Frt (purple triangles) and F3 (green triangles) are heterotypic target sequences for FLP recombinase; loxP (orange triangles) and lox5171 (yellow triangles) are heterotypic target sequences for Cre-recombinase; SA, splice acceptor; β-geo, β-galactosidase/neomycin phosphotransferase fusion gene; pA, bovine growth hormone polyadenylation sequence. [file pbio.2000197.s005.tif]

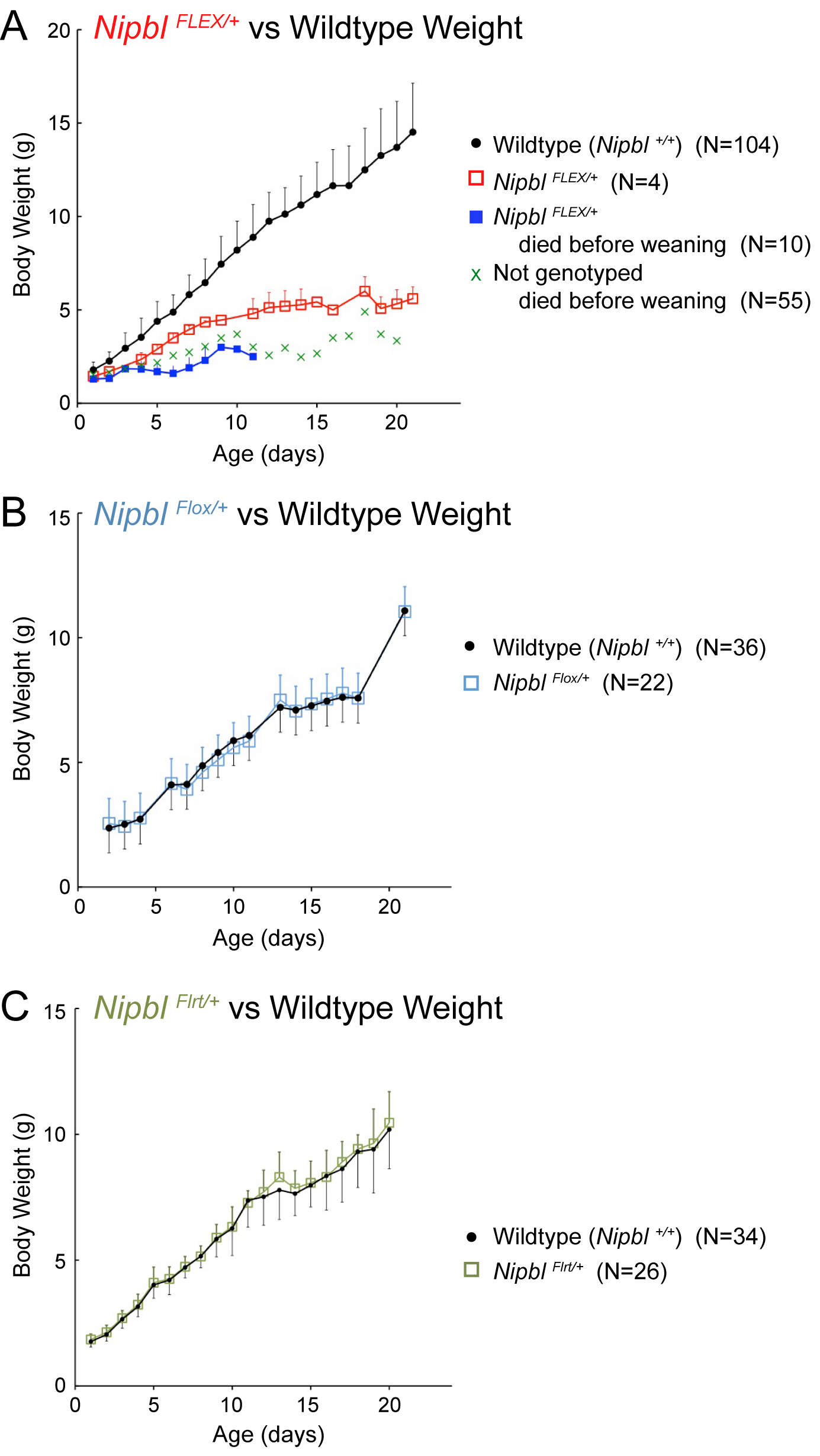

Supplement: S3 Fig — A. Growth curves of NipblFLEX/+ mice compared to wildtype littermates from post-natal day (P) 1 to P21, from 17 litters. Data are pooled by genotype (wildtype, NipblFLEX/+ or not genotyped due to early demise) and survival status (survived to weaning versus died before weaning), from birth to weaning at P21. For clarity, only upper error bars (standard deviation, SD) are shown for wildtype and NipblFLEX/+ daily weight averages. Data show that wildtype mice gain weight steadily for the first 21 days of life (black circles), whereas NipblFLEX/+ survivors (red open boxes) and non-survivors (blue filled boxes) grow at impaired rates. The weights of NipblFLEX/+ mice overlapped within the first few days of life regardless of whether they survived to weaning or not. Growth of un-genotyped non-survivors (green Xs) was similar to NipblFLEX/+ non-survivors (blue filled boxes) within the first 12 days of life, suggesting that pups that failed to survive were likely NipblFLEX/+. Some pups that were logged as having been born could not be genotyped because tissue could not be recovered. B. Growth curves of NipblFlox/+ mice compared to wildtype littermates from P1 to P21, from 5 litters. Data are pooled by genotype. Data show overlapping growth and similar sizes of wildtype and NipblFlox/+ mice. For purposes of clarity, only upper error bars for NipblFlox/+ mice (SD, blue) and lower error bars for wildtype (SD, black) are shown. C. Growth curves for NipblFlrt/+ mice compared to wildtype littermates from P1 to P21, from 4 litters. Data show overlapping growth and similar weights of wildtype and NipblFlrt/+ mice. For purposes of clarity, only upper error bars for NipblFlrt/+ mice (SD, green) and lower error bars for wildtype (SD, black) are shown. (TIF) [file pbio.2000197.s006.tif]

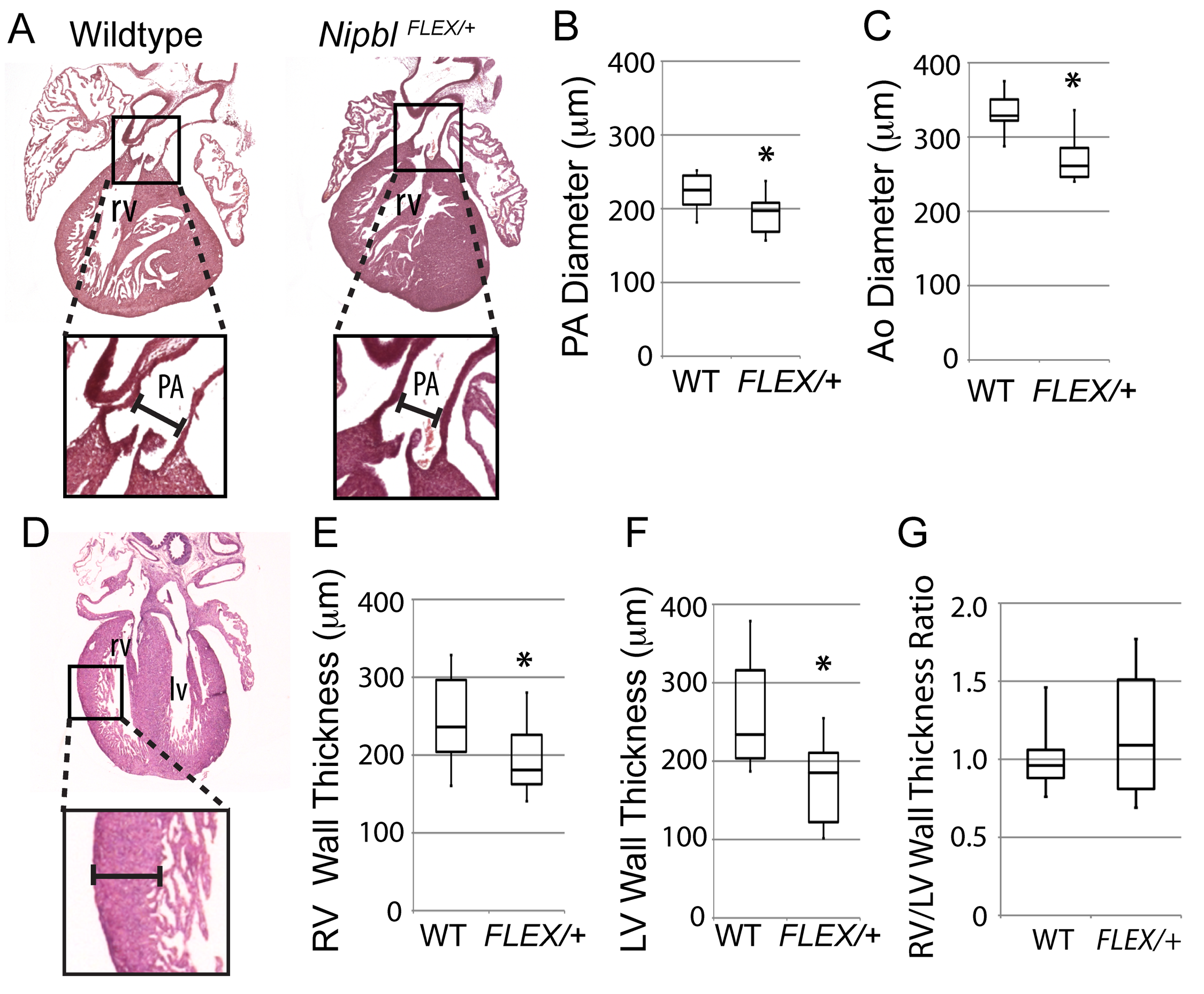

Supplement: S4 Fig — To assess stenosis, diameters of the aorta and pulmonary artery were measured from images using Axiovision software (Zeiss). 90% of pulmonary stenoses are isolated at the valvular region, so diameters were measured just distal to the semilunar valves (supravalvular). To confirm that any stenoses observed did not involve the pulmonary trunk/aorta, serial sections were followed by the observer to the bifurcation. To assess hypertrophic cardiomyopathy, ventricular wall thickness was measured and the ratio of the right to left wall thickness was calculated for each sample, and different genotypes were compared. Diameter of the ventricular wall (left and right myocardium, excluding trabeculae) was measured in images of H&E stained paraffin sections in the region that was midway through the atrioventricular valve (ventral-dorsal) and midway through the ventricular septum (superior to inferior). A. The supravalvular diameter of the great arteries of paraffin sectioned wildtype and NipblFLEX/+ hearts were measured as indicated by black bar. B. Both the pulmonary artery diameter and the aorta diameter (C) were significantly smaller in NipblFLEX/+ hearts (N = 11) compared to wildtype littermates (N = 9) (Mann-Whitney U, P<0.05). D. The thickness of the ventricular walls (excluding trabiculae) were measured as indicated by black bar. Ventricular wall thickness was significantly reduced in NipblFLEX/+ hearts (N = 11) compared to wildtypes (N = 9) on both the right (E) and left (F) walls (Mann-Whitney U, P<0.025). G. A ratio of the right to left ventricle wall thickness revealed no significant difference between wildtypes and NipblFLEX/+ mice (Mann-Whitney U P>0.05). Differences observed in C, D, E, and F likely due to the overall smaller body and heart size observed in NipblFLEX/+ embryos (see results and Fig 7). LV, left ventricle; PA, pulmonary artery; RV, right ventricle. (TIF) [file pbio.2000197.s007.tif]

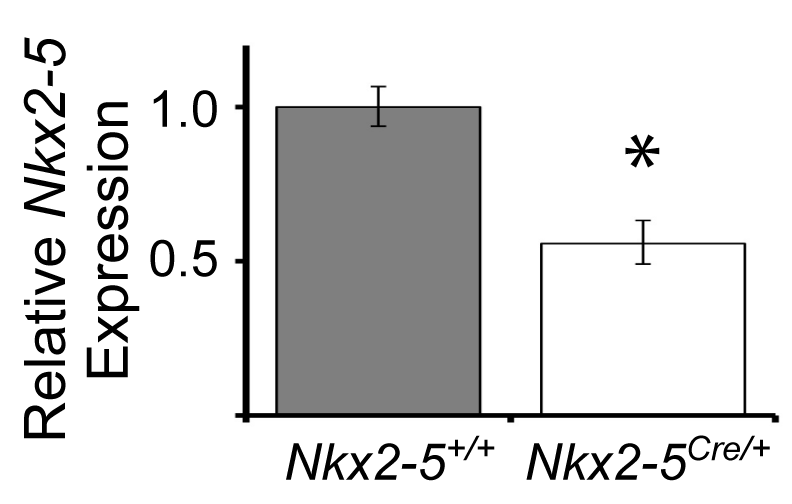

Supplement: S5 Fig — Q-RT-PCR was performed on E10.5 Nkx2-5+/+ (N = 7) and Nkx2-5Cre/+ (N = 5) hearts. Nkx2-5Cre/+ hearts had approximately half the Nkx2-5 expression of Nkx2-5+/+ littermates. Nkx2-5 mRNA expression was normalized to expression of B2m, and the relative expression of Nkx2-5 was obtained by the 2-ddCt method; data are expressed as mean ± SEM. PCR primers and conditions are described in Materials and Methods, and S1 Table. Asterisk: Student’s t-test, P = 0.001. (TIF) [file pbio.2000197.s008.tif]
